# Structure and Interactions of A Host Defense Antimicrobial Peptide Thanatin in Lipopolysaccharide Micelles Reveal Mechanism of Bacterial Cell Agglutination

**DOI:** 10.1038/s41598-017-18102-6

**Published:** 2017-12-19

**Authors:** Sheetal Sinha, Liangzhen Zheng, Yuguang Mu, Wun Jern Ng, Surajit Bhattacharjya

**Affiliations:** 1 School of Biological Sciences, Nanyang Technological University, 60 Nanyang Drive, Singapore, 637551 Singapore; 2 Advanced Environmental Biotechnology Centre, Nanyang Environment and Water Research Institute, Nanyang Technological University, 1 Cleantech Loop, Singapore, 637141 Singapore; 3 Division of Environmental and Water Resources, School of Civil and Environmental Engineering, Nanyang Technological University, 50 Nanyang Avenue, Singapore, 639798 Singapore; 4 Nanyang Environment and Water Research Institute (NEWRI), Nanyang Technological University, 1 Cleantech Loop, Singapore, 637141 Singapore; 5 Interdisciplinary Graduate School, Nanyang Technological University, 50 Nanyang Avenue, Singapore, 639798 Singapore

## Abstract

Host defense cationic Antimicrobial Peptides (AMPs) can kill microorganisms including bacteria, viruses and fungi using various modes of action. The negatively charged bacterial membranes serve as a key target for many AMPs. Bacterial cell death by membrane permeabilization has been well perceived. A number of cationic AMPs kill bacteria by cell agglutination which is a distinctly different mode of action compared to membrane pore formation. However, mechanism of cell agglutinating AMPs is poorly understood. The outer membrane lipopolysaccharide (LPS) or the cell-wall peptidoglycans are targeted by AMPs as a key step in agglutination process. Here, we report the first atomic-resolution structure of thanatin, a cell agglutinating AMP, in complex with LPS micelle by solution NMR. The structure of thanatin in complex with LPS, revealed four stranded antiparallel β-sheet in a ‘head-tail’ dimeric topology. By contrast, thanatin in free solution assumed an antiparallel β-hairpin conformation. Dimeric structure of thanatin displayed higher hydrophobicity and cationicity with sites of LPS interactions. MD simulations and biophysical interactions analyses provided mode of LPS recognition and perturbation of LPS micelle structures. Mechanistic insights of bacterial cell agglutination obtained in this study can be utilized to develop antibiotics of alternative mode of action.

## Introduction

**Figure 1:**
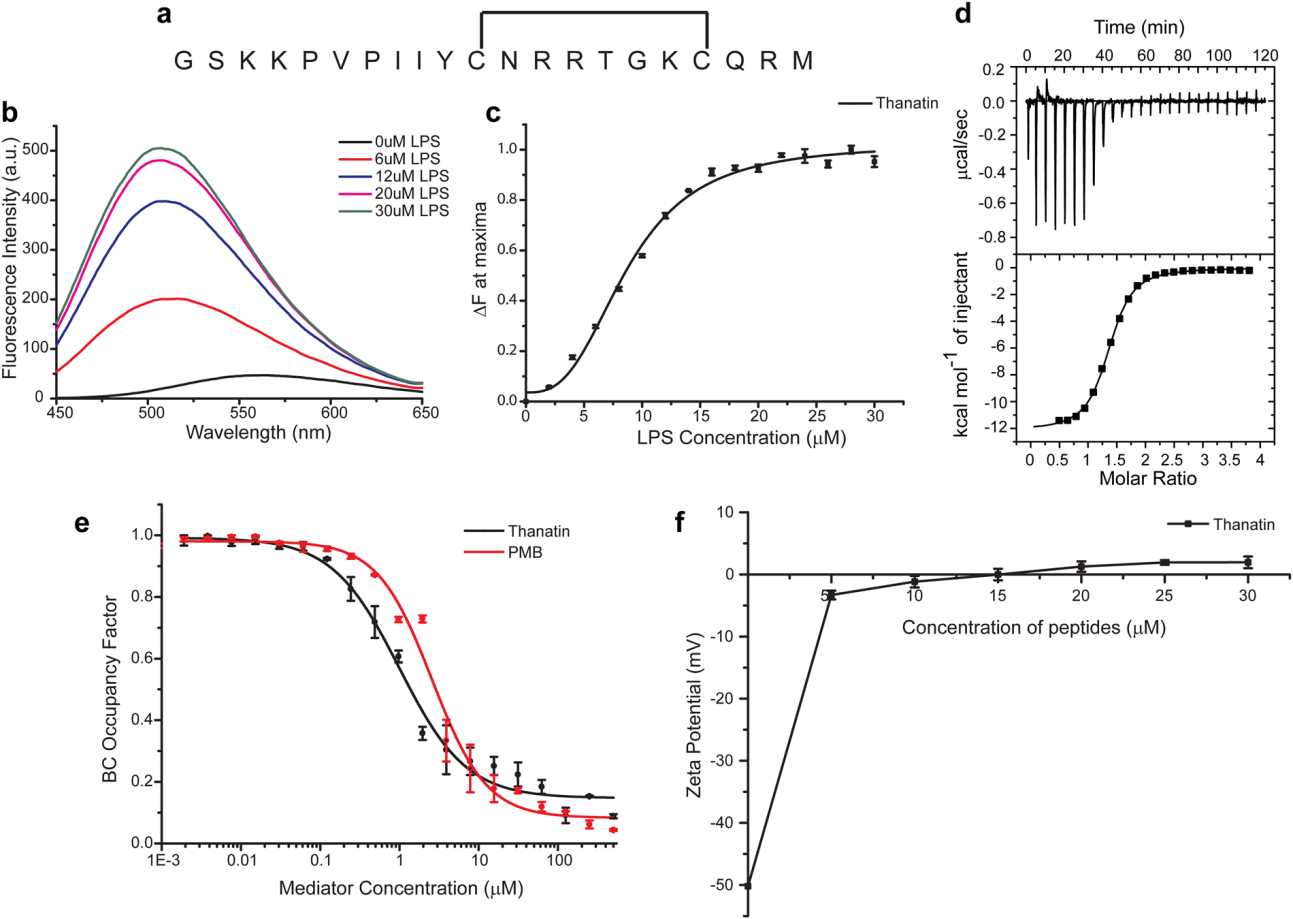
Thanatin interacts efficiently with outer membrane LPS. (**a**) Primary structure of thanatin. Thanatin contains a disulphide bridge between residues Cys 11 and Cys 18. (**b**) The fluorescence emission spectrum of dansyl group of dans-thanatin in presence of increasing LPS concentrations. Increase in fluorescence intensity and blue shift of the emission maxima in presence of higher concentrations of LPS indicated a less polar environment around the fluorophore. (**c**) Changes in fluorescence emission intensity of N-terminally dansylated thanatin in presence of varying concentrations of LPS reveal that thanatin/LPS interaction is cooperative in nature. (**d**) ITC thermogram reveals that thanatin/LPS binding is exothermic in nature. 1 mM stock of thatantin was titrated against 50 μM LPS in 10 mM sodium phosphate buffer, pH 7. (**e**) Comparison of BC probe displacement by thanatin and polymyxin B. A lower BC occupancy factor at a particular mediator concentration indicates a more efficient binding of the mediator to LPS. (**f**) Changes of zeta potential of *E. coli* cell solutions as a function of concentrations of thanatin.

Host defense cationic AMPs constitute the first line of defense against invading microorganisms. AMPs are known to clear infections from a broad range of pathogens including bacteria, viruses, parasites and fungi^1–3^. The recent occurrence of drug resistant and multiple drug resistant bacteria are global health threats^4,5^. The fight against drug resistant bacteria has been slowed down significantly due to the lack of new broad spectrum antibiotics^4,5^. The ability of AMPs to kill multiple drug resistant bacteria has been thought to be exploited for the development of novel antibiotics^1–3^. The membrane lytic activity of AMPs would cause difficulty for the emergence of resistance in bacteria^4,5^. As a mode of action, membrane active AMPs kill bacteria by pore formation in the plasma membrane by various mechanisms such as barrel stave model, toroidal pore model or by using an overall detergent-like action^6–9^. Mechanisms of such AMPs are extensively investigated in past and recent years. A class of AMPs, including several human proteins, exerts antibacterial activity by inducing bacterial cell agglutination^8–10^. Agglutinated cells can be efficiently removed by phagocytosis without releasing toxic substances into systemic circulation. In contrast to pore forming AMPs, cell agglutinating AMPs do not permeabilize cell membrane. Rather, as a mode of action, they directly interact with the outer membrane lipopolysaccharide (LPS) of Gram negative or cell wall peptidoglycans of Gram positive bacteria^10–13^. Atomic-resolution structures of cell agglutinating AMPs in complex with LPS or peptidoglycans would be essential to garner mechanistic insights in cell killing. However, 3-D structures of any of these AMPs in complex with LPS or peptidoglycans are yet to be reported. Thanatin is a 21-residue long inducible AMP (Fig. 1a) isolated from insect *Podisus maculiventris* after immune challenge^14–17^. Thanatin demonstrates antimicrobial activity against wide range of pathogens, including drug resistant bacteria at physiological concentrations^14–17^. It binds to the outer membrane LPS or cell wall peptidoglycan resulting in cell clumping or agglutination^14,17^. In order to understand cell agglutination mechanism of thanatin, we have investigated LPS/thanatin interactions and have determined its 3D structure in complex with LPS micelle, using an array of biophysical and NMR methods.

## Results

### Interactions and binding affinity of thanatin to LPS by fluorescence, isothermal titration calorimetry (ITC) and zeta potential measurements

The primary structure of thanatin does not contain an intrinsic fluorophore e.g. Trp, therefore, N-terminal dansylated thanatin or dans-thanatin was utilized for interactions of thanatin with *E. coli* 0111:B4 LPS. The fluorescence emission spectrum of dansyl group of dans-thanatin in free solution was of low intensity with a λ_max_ ~ 560 nm (Fig. 1b). The emission spectra of dans-thanatin demonstrated enhanced intensity and a concomitant blue shift in λ_max_, to ~510 nm at 30 μM LPS, upon additions of increasing concentrations of LPS (Fig. 1b). The λ_max_ value of the dansyl group indicated that it is plausibly located at the LPS micelle-water interface. A similar λ_max_ value of dansyl group was reported while the probe has been found to be localized at the membrane-water interface^18^. The intensity enhancement (ΔF at λ_max_) of dans-thanatin has been plotted as a function of LPS concentration yielding at apparent equilibrium dissociation constant K_d_ ~ 8.6 ± 0.38 μM (Fig. 1c). LPS-thanatin interactions were further analyzed using isothermal titration calorimetry (ITC) experiments estimating binding affinity and thermodynamic parameters (Fig. 1d). LPS-thanatin interactions were observed to be exothermic in nature as suggested by downward position of titration peaks and integrated heat values (Fig. 1d). ITC derived LPS-thanatin binding parameters are provided in Table 1. As seen, thanatin binds to LPS with K_d_ ~ 1.55 μM and binding is essentially favoured by negative enthalpy (ΔH) change (Table 1). Note, a somewhat higher K_d_ value of thanatin-LPS interactions was estimated from the dansyl fluorescence changes compared to ITC. The difference may arise from the type of interactions determined by the two different methods. Dansyl fluorescence probes only localized changes whereas ITC measures more global interactions. Next, we examined ability of thanatin to displace BODIPY-cadaverine (BC), a fluorescence probe from LPS. BC binds to the lipid A domain of LPS through ionic interactions between protonable amine groups of the probe and anionic phosphates of lipid A^19,20^. BC probe displacement assays demonstrated that thanatin binds efficiently to LPS, with an estimated ED_50_ of 0.87 ± 0.15 μM (Fig. 1e). BC displacement was also carried out for polymyxin B, an archetype LPS binding peptide that displayed a somewhat higher ED_50_ of 3.7 ± 0.30 μM (Fig. 1e). Surface charge neutralization of *E. coli* was examined by measuring zeta potential as a function of concentrations of thanatin (Fig. 1f). The anionic LPS-outer membrane predominantly contributes to a negative zeta potential of *E. coli* cells^21–23^. As expected, addition of thanatin to *E. coli* cell solutions, at various doses, caused a dramatic increase in zeta potential toward a neutral surface potential (Fig. 1f). Even at 5 μM of thanatin, zeta potential changed from −50 mV to −5 mV and the surface charge effectively became neutral (zeta potential ~0 mV) at a peptide concentration of 15 μM (Fig. 1f). These data suggests that thanatin binds to *E. coli* cells utilizing ionic interactions among cationic residues and anionic membranes. The aforementioned data, BC probe displacement ITC and zeta potential, thus demonstrated that thanatin-LPS interactions could be largely dominated by ionic interactions. Although, dans-thanatin fluorescence data suggested that thanatin also inserts into the hydrophobic milieu of LPS.

**Table 1:** ITC derived binding parameters of thanatin titrated against 50 μM LPS in 10 mM phosphate buffer, pH 7, at 298 K.

	K_d_ (μM)	ΔH (Kcal/mol)	TΔS (Kcal/mol)	ΔG (Kcal/mol)
**Thanatin**	1.55	−12.16	−4.24	−7.92

### Structure of thanatin in free solution by NMR

**Figure 2:**
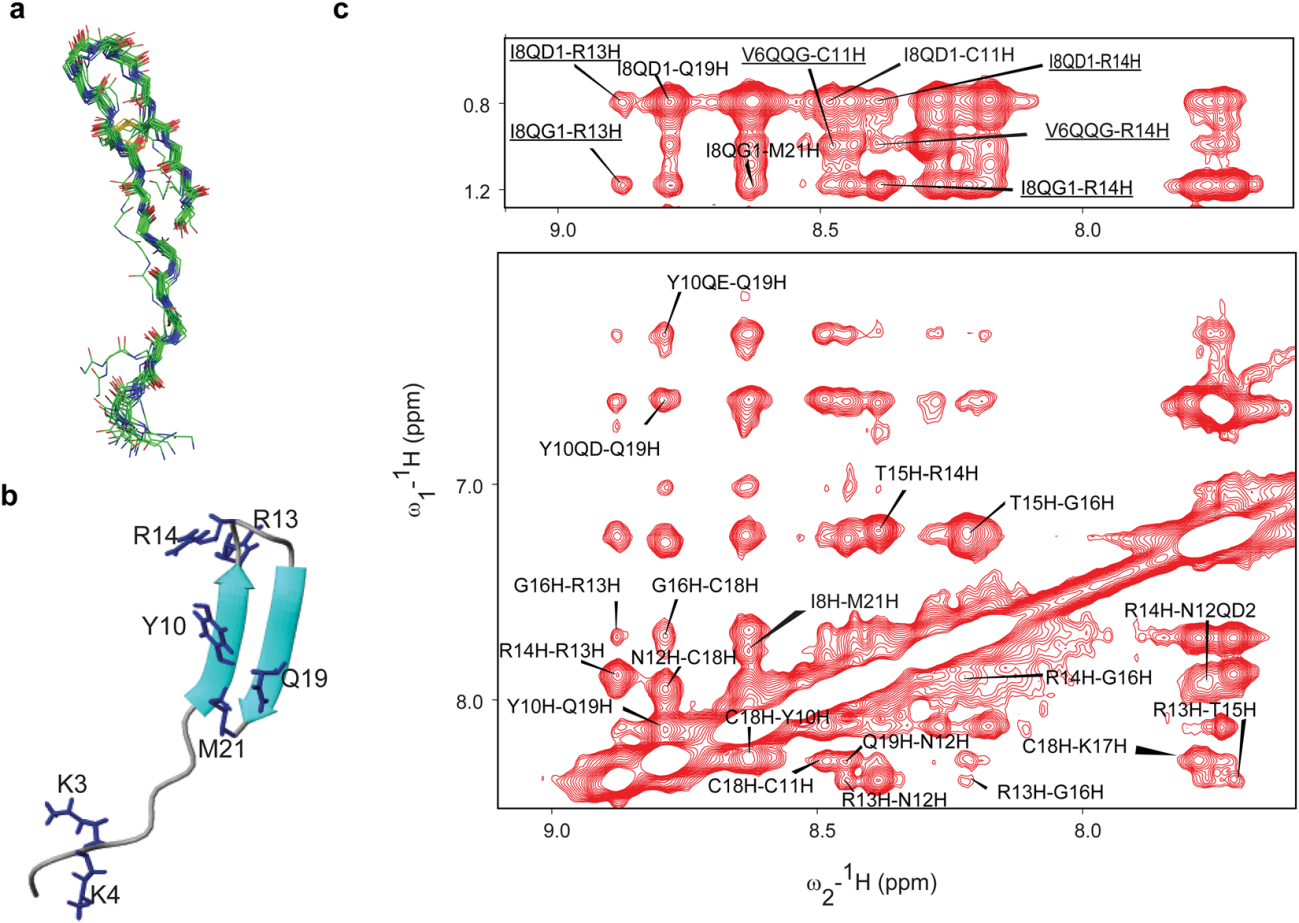
Thanatin assumes monomeric β-sheet structure in free solution and displays tr-NOEs in LPS micelle. (**a**) Superposition of backbone atoms of twenty lowest energy structures of thanatin. It assumes a monomeric β-hairpin structure with an extended N-terminus in free solution at 25 °C, pH 5. (**b**) Representative structure of thanatin in free solution. (**c**) Sections of two dimensional tr-NOESY spectrum of thanatin, in presence of LPS, showing NOE connectivity among amide proton resonances (ω2 dimension) with amide/aromatic proton resonances and aliphatic proton resonances (ω1 dimension). Some unique NOE cross peaks (underlined) which are present only in tr-NOESY spectrum of thanatin/LPS complex are incompatible to the β-hairpin structure of monomeric thanatin and are used as inter-monomeric NOEs for structure determination.

3-D structure of thanatin in free solution has been previously reported in an aqueous solution at a low temperature of 5 °C by using NMR spectoscopy^24^. In order to realize thanatin structure and interactions in LPS, we have analysed NMR spectra, two-dimensional ^1^H-^1^H TOCSY and NOESY, and determined solution structure of thanatin in aqueous solution at 25 °C. An ensemble of structures, in free solution, of thanatin was determined based on 140 NOE driven distance constraints and dihedral angle constraints (Fig. 2a). As can be seen, thanatin assumes an extended conformation for N-terminal residues G1-P7 followed by a beta hairpin like structure containing two stranded antiparallel β-strands, residues I8-N12 and residues K17-M21, connected by a loop comprising residues R13-G16 (Fig. 2b). The β-hairpin structure is stabilized by a single disulfide bond involving residues C11 and C18 (Fig. 2b). The 3-D structure of thanatin determined here at 25 °C is akin to the structure previously determined at 5 °C^24^.

### NMR analyses of thanatin in LPS micelle

Transferred nuclear Overhauser effect spectroscopy (tr-NOESY)^25,26^ can be utilized to determine atomic-resolution structures of AMPs and LPS neutralizing peptides in LPS micelle^27–33^. Owing to the low critical micelle concentration (<1 μM), LPS readily forms high molecular weight micelles in solution^34,35^. AMPs undergoing chemical exchange at the time scale of fast or intermediate exchange between free and LPS bound states may yield tr-NOEs permitting determination of bound conformations^27–33^. Additions of LPS, from a stock solution, into a solution containing thanatin, showed broadening of resonances in one-dimensional NMR spectra (Supplementary Fig. 1). Such an NMR line broadening effect of a ligand upon addition of large macromolecules can be interpreted as either fast or intermediate chemical exchange^25,26^. Fast or intermediate time scale of chemical exchange between thanatin and LPS micelles has been further supported by the observation of tr-NOEs. Identification of amino acid spin systems or sequence specific resonance assignment of thanatin in LPS micelles were carried out by combined analyses of TOCSY and NOESY spectra (Supplementary Figs 2 and 3). Chemical shift of amino acids of thanatin has been provided in Supplementary Table S1. Two-dimensional tr-NOESY spectra of thanatin, in presence of LPS micelle, yielded more number of NOE connectivities in comparison to thanatin in free solution involving sidechain/sidechain and backbone/sidechain resonances (Supplementary Fig. 4). Moreover, there were several new long range NOEs detected for residues, V6, I8, Y10, R14, K17 of thanatin in LPS micelles (Supplementary Fig. 5). Notably, residue Y10 demonstrated as many as ~40 NOEs in complex with LPS micelle (Supplementary Fig. 5). Interestingly, a number of unique long-range NOEs were detected among residues e.g. V6/C11, I8/N12, I8/R13, I8/R14 for thanatin in complex with LPS (Supplementary Table S2, Fig. 2c). These NOEs demonstrated a close proximity among residues at the extended N-terminus and residues at the β-turn of the β-sheet structure of thanatin.

### 3-D structure of thanatin in LPS micelle

**Figure 3:**
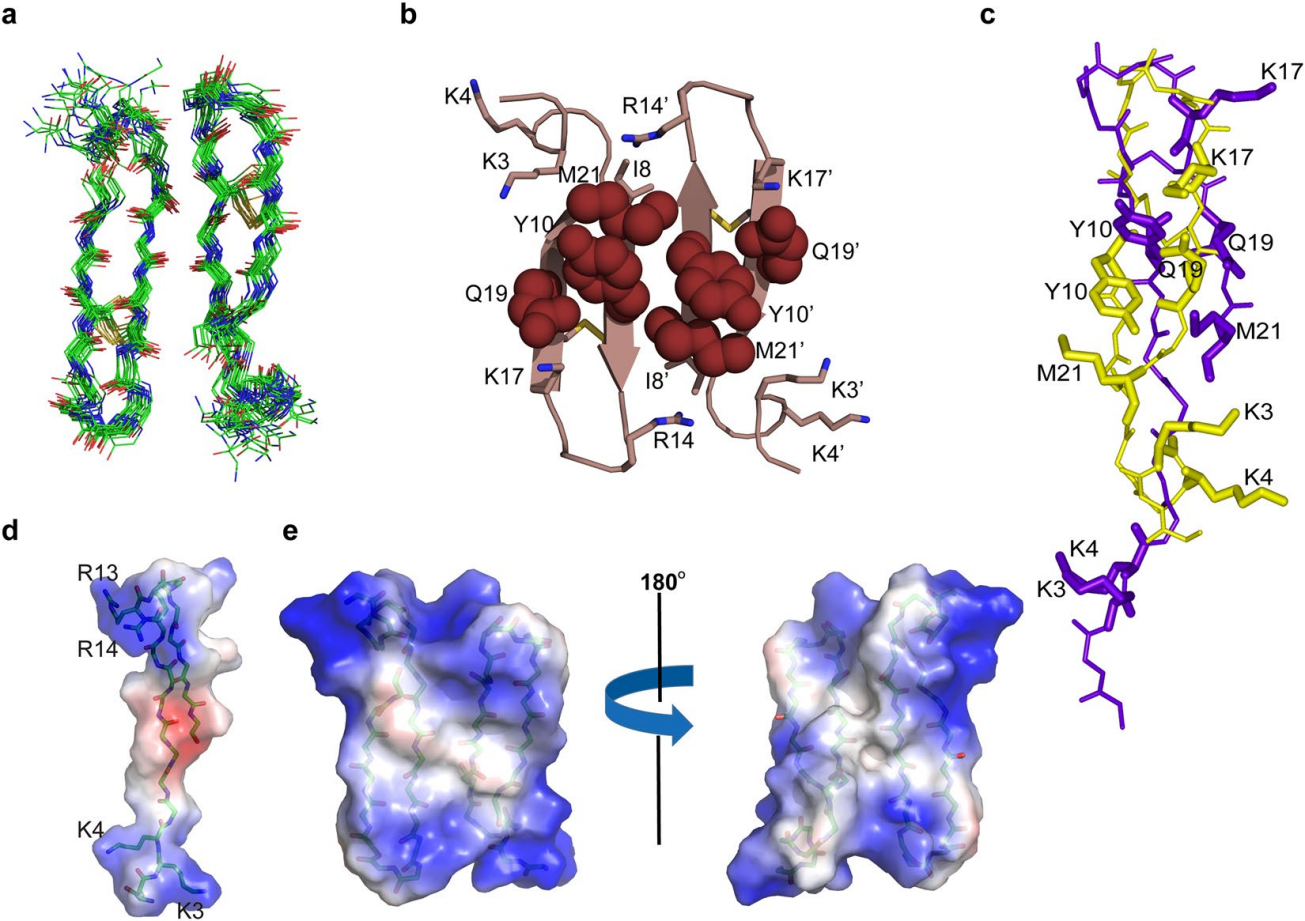
Thanatin assumes dimeric structure in complex with LPS micelle. (**a**) Superposition of backbone atoms of twenty lowest energy dimeric structures of thanatin as a complex of LPS micelle. The long N-termini regions of the two monomers are oriented in an anti-parallel fashion. (**b**) Ribbon representation of the dimeric structure of thanatin showing packing residues (in space fill mode) and sidechains of cationic residues (in stick). (**c**) Superposition of backbone atoms of the one sub-unit of thanatin dimer (yellow) with monomer structure determined in free solution (violet). An RMSD of 5.1 Å for backbone atoms and 6.2 Å for heavy atoms were estimated. (**d**) Electrostatic surface potential of monomeric thanatin in free solution and (**e**) dimeric thanatin in LPS micelle.

Figure 3a shows superposition of backbone atoms (Cα, N and C’) of twenty low energy structures of dimeric thanatin in complex with LPS. NOE driven distance constrains for residues V6/C11, I8/N12, I8/R13, I8/R14 (Supplementary Table S2) were found to be incompatible to a monomeric β-sheet structure and were utilized as inter-monomeric or dimeric NOEs for structure determination. The ensemble of dimeric structures of thanatin was determined utilizing 386 NOE driven distance constrains including 78 long range and 10 inter subunit NOEs (Supplementary Table S3). The structural statistics and RMSDs are provided in Supplementary Table S3. The 3-D structure of dimeric thanatin in complex with LPS revealed a four stranded β-sheet topology, whereby the residues at the long N-terminal region (residues 1–13) of the two monomers are engaged in a non-covalent interface in an antiparallel fashion. The two subunits of the dimer sustained close packing interactions between sidechains of aromatic and hydrophobic residues, Y10/Y10′, M21/M21′ and Q19/Q19′ (Fig. 3b). Further, a number of cationic residues, namely R13, R14 at the β-turn region and K3 and K4 at the N-terminus, in the dimeric structure of thanatin are situated in spatially proximal orientation (Fig. 3b). Figure 3c shows the superposition of structures of free thanatin and in complex with LPS (for one of the subunit). As seen, there are significant differences between the LPS bound structure and free structure of thanatin both in backbone and sidechain dispositions. The RMSD values for backbone atoms and all heavy atoms were estimated to be 5.1 Å and 6.2 Å, respectively. Strikingly in complex with LPS micelle, the cationic N-terminal segment of thanatin comprising residues G1-P7, acquires a define conformation and folds back toward the β-hairpin structure. In particular, residues K^3^KPV^6^ adopt a β-turn conformation centring residues K4 and P5 at i + 1 and i + 2 positions, respectively (Supplementary Fig. 6). The electrostatic potential surfaces of dimeric and monomeric thanatin are shown in Fig. 3. The dimeric structure of thanatin in LPS demonstrates large cationic patches at the distal ends and an extended non-polar surface at the centre of the four stranded β-sheet. Therefore, in comparison to the monomeric structure, the dimeric structure of thanatin possesses higher cationicity (12.8 vs 7) and exposed hydrophobic surface (2203 Å^2^ vs 1603 Å^2^) amenable for LPS interactions.

### Interactions of thanatin with LPS by Saturation Transfer Difference (STD) NMR

**Figure 4:**
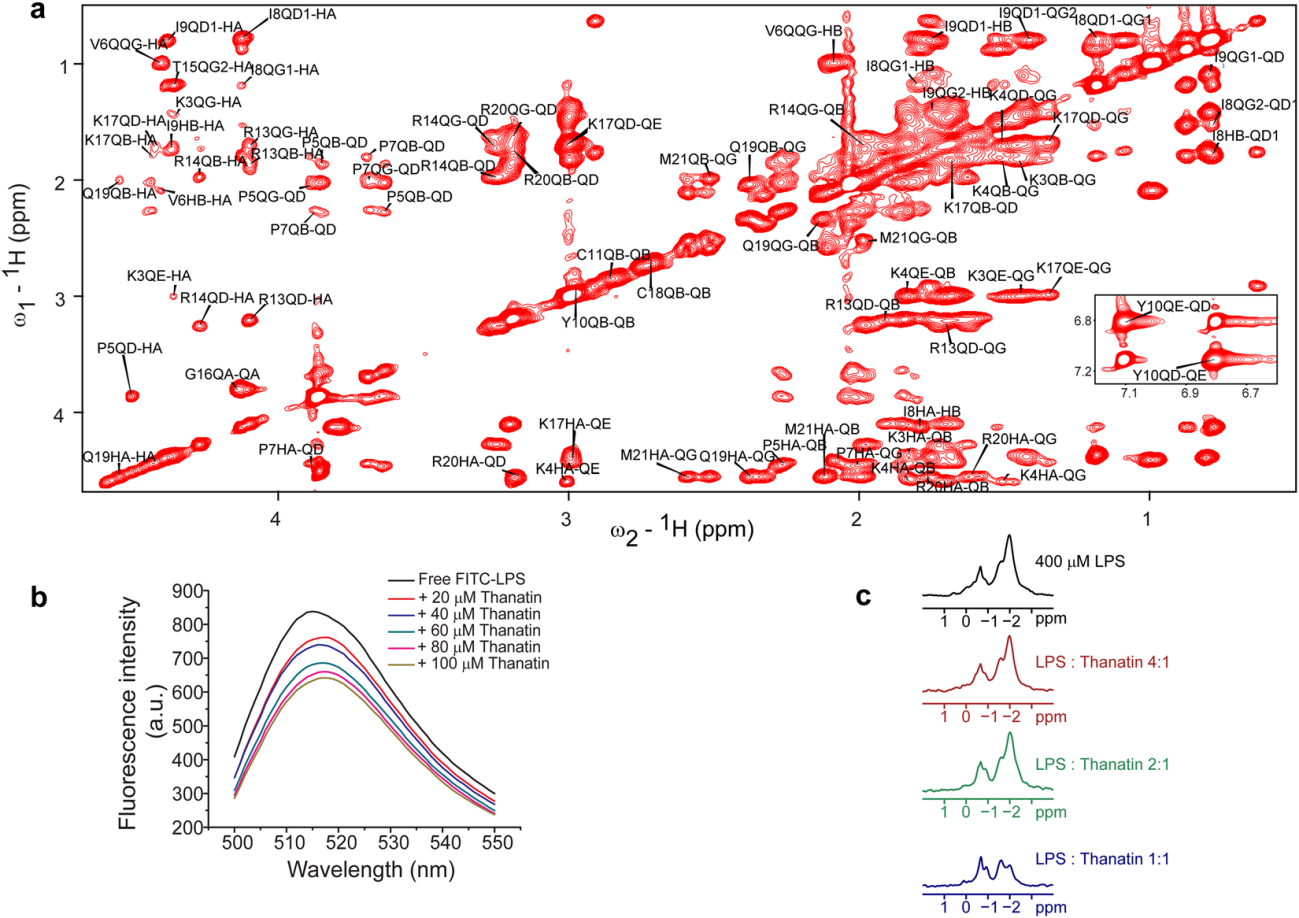
Thanatin binds intimately to LPS micelles and causes micelle aggregations. (**a**) Two-dimensional STD-TOSCY spectrum of thanatin in presence of LPS micelle in D_2_O showing STD correlations among upfield shifted resonances. (inset) STD from downfield shifted aromatic ring proton resonances of residue Y10. (**b**) Fluorescence emission spectra of FITC-LPS at different concentrations of thanatin. Unlike other pore forming AMPs, emission intensity of FITC-LPS quenches with increasing concentrations of thanatin. (**c**) ^31^P NMR spectra of LPS at various molar ratios of thanatin. Extensive line broadening indicates interactions of phosphate groups of LPS with thanatin.

STD-NMR method can map contact residues of ligands in complex with receptor molecules at an atomic resolution within a fast chemical exchange regime^36–38^. STD-NMR has been utilized to determine contact residues of potent pore forming AMPs in LPS micelle^39–42^. One-dimensional ^1^H STD and two-dimension ^1^H-^1^H STD-TOCSY experiments were carried out to identify residues of thanatin in contact with LPS micelle (Supplementary Fig. 7). Due to better resolution, two-dimension STD-TOCSY spectra were analyzed identifying residues of thanatin in contact with LPS micelle (Fig. 4a). The STD-TOCSY spectrum of thanatin showed cross-peaks arising from CαHs to sidechains and among sidechain/sidechain resonances for a number of residue (Fig. 4a). As reported in earlier works, resonances demonstrating cross-peaks in the STD-TOCSY spectra are supposed to be receiving a high degree of saturation from the binding partners due to their close proximity^36–38^. STD-TOCSY correlations appear to be more intense among the sidechains resonances compared to that of backbone CαH to sidechain resonances, indicating closest proximity of sidechains to LPS micelle (Fig. 4a). In particular, conspicuous STD-TOCSY correlations could be detected for the basic residues including K3, K4, R13, R14, K17 and R20, indicating their proximity with LPS micelle. Residues P5, V6, P7 also delineated STD effect. Note, these N-terminal residues K3, K4, P5 and V6 have experienced large conformational change in complex with LPS micelle. By contrast, among the polar residues, (S2, N12, C11, T15, C18, and Q19), residues N12, T15 and Q19 showed STD-TOCSY correlations. The single aromatic residue Y10 displayed strong STD effect involving ring protons and CαH/CβH protons, indicating its association with LPS micelle (Fig. 4a (inset), Supplementary Fig. 7). Note, although residue Y10 is engaged at the dimeric interface of thanatin, calculation of solvent accessible surface area showed that the sidechain of residue Y10 is not completely buried and remains amenable for interactions with LPS micelles. The non-polar sidechains of residues I8, I9 and M21 remain in a close contact with LPS micelle as demonstrated by the strong STD-TOCSY correlations. Taken together, STD analyses suggest that most of the residues, including cationic, aromatic and non-polar, of thanatin are essentially in contact with LPS micelle.

### Effect of thanatin on LPS micellar structure

We have examined structural changes of LPS micelles in complex with thanatin using FITC labelled LPS or FITC-LPS and ^31^P NMR analyses. Emission intensity of FITC-LPS is quenched due to FRET among the fluorophore in an aggregated LPS micelle structure^43–45^. Dissociation of LPS micelle upon binding to proteins or pore forming AMPs often results in an enhancement of FITC emission intensity^44,45^. Note, in FITC-LPS conjugate, FITC is covalently linked with the sugar residues located at the solvent permeable surface of LPS^46,47^. Therefore, FITC fluorescence change would probe the LPS surface interactions with peptides. Figure 4b shows fluorescence emission spectra of FITC-LPS at various concentrations of thanatin, ranging from 0 to 100 μM. As seen, emission intensity of FITC-LPS demarcated a marked quenching with increased concentrations of thanatin (Fig. 4b). The diminution of emission intensity of FITC-LPS in complex with thanatin, indicated a higher FRET among the fluorophore FITC. In other words, FITC-LPS fluorescence data demonstrate that LPS micelles undergo a higher order association upon binding with thanatin. Perturbation of LPS micelle structure by thanatin was further probed from ^31^P NMR experiments. ^31^P NMR spectra of LPS were acquired in free solution and at three different molar ratios, 4:1, 2:1 and 1:1 of LPS:thanatin (Fig. 4c). The free LPS micelles produces two well resolved NMR signal at ~−1.00 ppm and −2 ppm, respectively^39,44^. Both the phosphate groups demonstrated extensive line broadening when in complex with thanatin, indicating involvement of the phosphate head groups in binding with thanatin (Fig. 4c). As evident, at higher concentration of thanatin, at 1:1 molar ratio, ^31^P NMR signals of LPS appeared to be extremely broadened. Such resonance broadening effect may potentially arise from slower tumbling motion of aggregated LPS micelles in complex with thanatin. Taken together, the aforementioned experiments, FITC-LPS fluorescence and ^31^P NMR indicated that the LPS micelles undergo structural rearrangements or an association to larger LPS aggregates in complex with thanatin.

### Modelling of LPS-dimeric thanatin complex by Molecular Dynamics (MD) simulations

**Figure 5:**
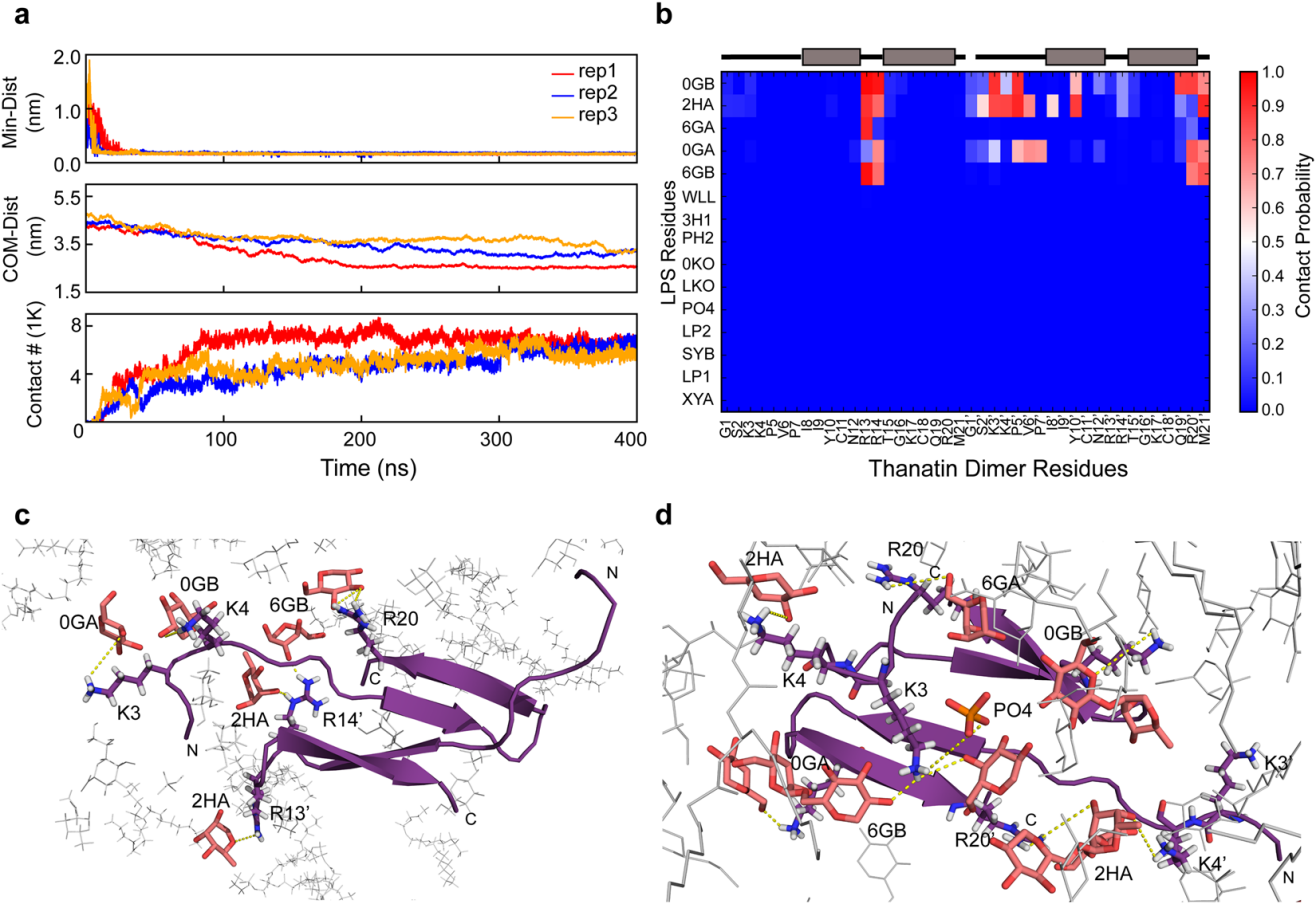
MD simulations revealed dimeric thanatin interact with the head group of multiple LPS. (**a**) Time revolutions of thanatin-LPS minimum distances, center of mass distances (in z direction) and the number of contacts in LPS/DPPE bilayer during MD simulations reveal that dimeric thanatin rapidly binds to LPS lipid surface. (**b**) Contact plot showing probability of each residue in dimeric thanatin with various LPS residues indicates that N terminal residues interact with the sugar rings of LPS molecules. The secondary structures of thanatin have been shown at the top of the panel, thick bars (in light black) represent β-strands whereas other structural elements are in thin black line. (**c** and **d**) Two different binding modes of dimeric thanatin with LPS molecules in LPS/DPPE bilayer. (**c**) Binding mode I: dimeric thanatin interacts with LPS molecules utilizing residues at the N terminus of one subunit and residues in the turn from another subunit. (**d**) Binding mode II: dimeric thanatin interacts with LPS molecules employing residues from both the N terminal tails.

In order to gain further insights into LPS-thanatin interactions, MD simulations were carried out for 3 repeats of 400 ns each in LPS/DPPE bilayer and dimeric thanatin (see Materials and Methods). The LPS/DPPE bilayer may be considered as a mimic of outer membrane of Gram negative bacteria where the outer-leaflet of the outer membrane is predominantly occupied by LPS molecules. Figure 5 summarises MD simulations of dimeric thanatin and LPS/DPPE bilayer. Figure 5a shows that interactions between dimeric thanatin and LPS lipids during MD simulations revealed a rapid, within 40–50 ns, encountering and stabilization of the LPS-thanatin complex. Both the subunits of the dimeric thanatin have been found to form a network of interactions with multiple LPS molecules, primarily with the head regions, through multiple electrostatics, polar and van der Waals contacts (Fig. 5b,c and d). The dimeric thanatin latches onto the LPS surface following two different binding modes. In binding mode I, residues at the N terminal tail from one subunit and cationic residues R13 and R14 from the middle loop of another subunit appeared to establish interactions with LPS molecules (Fig. 5b and c). As seen, residues R13 and R14 form multiple hydrogen bonds with hydroxyl groups of LPS (Fig. 5c). Moreover, residues, P5′, V6′, P7′ and I8′ interact with the sugar rings of LPS through non-polar contacts (Fig. 5b). In the binding mode II, both the N terminal tails of the dimer are engaged in interactions with LPS surface (Fig. 5d, Supplementary Fig. 8). The basic residues K3′ and K4′ of the N terminal tail in the dimeric thanatin establish electrostatic interactions with LPS phosphate groups (Fig. 5d).

### Ala scanning mutations of cationic and hydrophobic residues of thanatin

**Figure 6:**
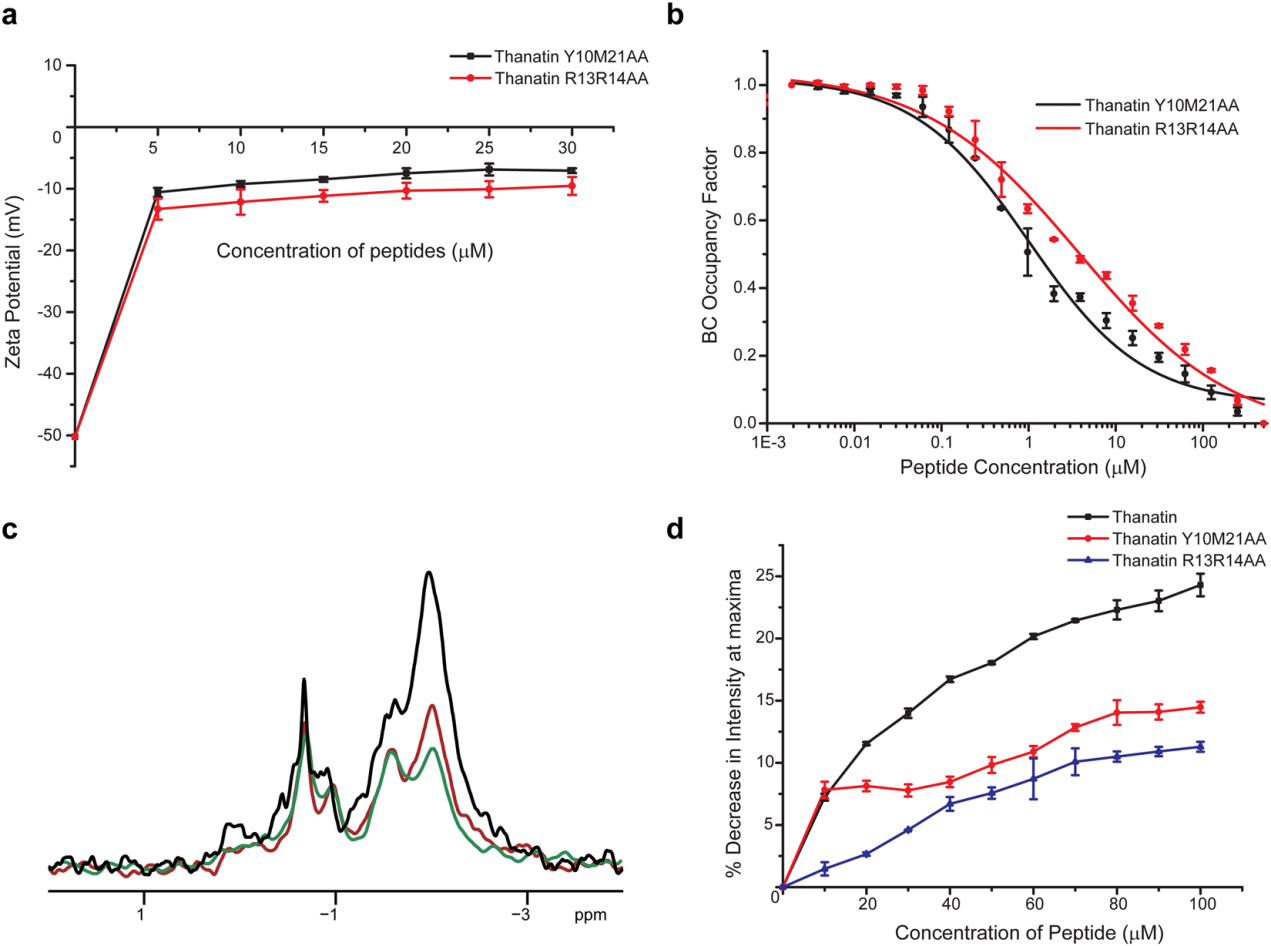
Alanine mutations of critical residues of thanatin diminish interactions with LPS. (**a**) Zeta potential measurements of *E. coli* with increasing concentrations of thanatin analogs. Data show that the analogs were not able to neutralize the negative zeta potential effectively. (**b**) BC probe displacement by the analogs peptides Y10M12AA and R13R14AA from LPS micelles. A much higher ED_50_ values were estimated compared to that of native thanatin suggesting a diminished LPS binding affinity. (**c**) ^31^P NMR spectra of LPS in complex with thanatin analogs demonstrated reduced broadening of ^31^P NMR signals, compared to native thanatin, at 1:1 molar ratios. ^31^P NMR spectra of free LPS (black), in presence of analogs R13R14AA (in red) and Y10M12AA (in green). (**d**) Changes in fluorescence emission intensity at λ_max_ of FITC-LPS with increased concentrations of thanatin and its analogs demonstrated limited changes caused by the analogs as compared to the native thanatin.

In order to correlate LPS-dimer thanatin structures and activity, critical residues Y10, M21, R13 and R14 of thanatin, involved in potential LPS recognition, were mutated and antimicrobial activities and LPS interactions were determined. We hypothesized that these analogs with double replacements may generate a larger difference in LPS binding and antimicrobial activity compared to single mutants. As seen, replacements of aromatic/hydrophobic packing residues Y10 and M21 to Ala had substantially decreased antimicrobial activity as indicated by higher MIC values of the analog peptide Y10M21AA. On the other hand, the R13R14AA analog was found to be largely inactive (Table 2). Figure 6a shows dose dependent changes in zeta potential of *E. coli* cells by peptides Y10M21AA and R13R14AA. Although, additions of mutant peptides caused surface charge neutralization toward lower negative values, however, complete surface charge neutralization was not observed even at the highest concentration of peptides (30 μM). Therefore, unlike native thanatin, mutant analogs are less efficient in complementing surface charge of bacterial cells. We further estimated LPS binding of the mutant analogs by BC probe displacement studies. Figure 6b shows BC probe displacement of the mutant analogs Y10M21AA and R13R14AA. The ED_50_ for Y10M21AA and R13R14AA peptides were estimated to be 1.22 μM and 3.20 μM, respectively. Note, the native thanatin demonstrated an ED_50_ of 0.87 μM in probe displacement (Fig. 1d). These data indicate that the mutant analogs bind to LPS with lower affinity compared to the native thanatin. In particular, LPS binding affinity appears to be more diminished for R13R14AA analog. Perturbations of LPS micelle structure by analog peptides were further investigated. ^31^P NMR studies of LPS demonstrated sustained intensity of ^31^P signals after additions of Y10M21AA and R13R14AA analogs (Fig. 6c). Further, FITC-LPS fluorescence spectra were obtained for the analog peptides. As seen, additions of analog peptides into FITC-LPS solutions had yielded limited changes in FITC fluorescence intensity, compared to the native thanatin (Fig. 6d). The aforementioned investigations clearly established that the analog peptide R13R14AA substantially lacked bactericidal activity whereas antibacterial activity of Y10M12AA peptide has also been reduced (Table 2). These peptides were inefficient in bacterial surface charge neutralization. Furthermore, these analog peptides possess lower binding to LPS and were unable to generate structural changes to the LPS structures.

**Table 2:** Minimum inhibitory concentration (µM) of thanatin and its analogs against EC: *Escherichia coli*; PA: *Pseudomonas aeruginosa*; SE: *Salmonella enterica*; KP: *Klebsiella pneumoniae*; SA: *Staphylococcus aureus*; BS: *Bacillus subtilis*; SP: *Streptococcus pyogenes*; EF: *Enterococcus faecalis*.

Peptide	Peptide Sequence	MIC (μM)							
		Gram Negative				Gram Positive			
		EC	PA	SE	KP	SA	BS	SP	EF
Thanatin	GSKKPVPIIYCNRRTGKCQRM	0.5	1	1	1	1	2	0.5	0.5
Thanatin Y10M21AA	GSKKPVPIIACNRRTGKCQRA	6	4	4	2	4	8	6	2
Thanatin R13R14AA	GSKKPVPIIYCNAATGKCQRM	>100	>100	>100	>100	>100	>100	>100	>100

## Discussion

The outer member LPS plays important roles in survival of Gram negative bacteria creating a permeability barrier against antibiotics and bactericidal agents^48–50^. Endotoxin function of LPS is also known to cause fatal septic shock syndromes in humans and other animals^51–53^. At present, there are no drugs of therapeutic modality against sepsis or septic shock^54,55^. LPS being a strong inflammatory molecule is also associated with other diseases^56,57^. Therefore, atomic-resolution structures and interactions analyses of AMPs in complex with LPS would not only yield mechanistic insights for bacterial outer membrane disruption, but structural scaffolds can be utilized for the generation of antibiotics and endotoxin neutralization drugs^58–60^. Towards this, 3-D structures of several pore forming AMPs in complex with LPS have been reported in past and recent years^39–42^. Opposed to pore formation which requires AMPs to disrupt inner membrane, bacterial cell agglutinating AMPs target outer membrane components^10,11,17,19^. Bacterial cell killing by agglutination has been described for a number of antimicrobial peptides, proteins and recently for amyloid Aβ peptides^61–63^. However, the mechanism of cell agglutination is yet to be elucidated. One of the key steps in cell agglutination of Gram negative bacteria is the binding of AMPs with the outer membrane LPS. In order to glean knowledge in cell agglutination process, we have determined the first 3-D structure of a host defence cell agglutinating AMP, thanatin, in complex with LPS micelle and investigated its interactions. Biophysical and optical spectroscopic studies demonstrated characteristic interactions and estimated thanatin-LPS binding parameters. Dansyl fluorescence emission of dans-thanatin indicated potential inclusion of thanatin into non polar environment of LPS micelles (Fig. 1b and c). On the other hand, exothermic binding interactions suggested that LPS-thanatin complex may be stabilized by ionic and/or hydrogen bonding (Fig. 1d, Table 1). Whereas, negative entropy change appears to suggest more ordering of the LPS-thanatin complex (Table 1). Further, thanatin mediated efficient release of BC probe from LPS (Fig. 1e) and *E. coli* surface charge neutralization (Fig. 1f) indicated predominant ionic interactions with the lipid A head groups and outer membrane LPS, respectively. STD-NMR analyses demonstrated that thanatin has been intimately associated with LPS micelle utilizing many of the cationic and non-polar and aromatic residues (Fig. 4a). Further, binding of thanatin into LPS micelle yielded changes of LPS micellar structure, towards lager aggregates, as observed from ^31^P NMR and FITC-LPS fluorescence studies (Fig. 4b and c). Such interactions and complex formation between LPS-thanatin would contribute to the outer membrane binding and cell agglutination process.

In order to better correlate, atomic resolution structures of thanatin were determined in free solution and in complex with LPS micelle. Free thanatin assumed a β-hairpin structure for the central sequence (residues I8-M21) along with an N-terminal extended region (Fig. 2a and b). By contrast, thanatin in complex with LPS micelles demonstrated a higher order structural assembly of a dimeric four stranded β sheet (Fig. 3). Notably, 3-D structures of pore forming β-sheet antimicrobial peptides in complex with LPS have been determined^64–66^. These structures revealed monomeric topology of β-sheet AMPs which may be necessary for outer membrane permeabilization followed by traversing to the bacterial plasma membrane As seen, the dimeric structure of thanatin has resulted in close proximity of the N-terminal β-strand of one subunit and N-terminal β-strand of another subunit in antiparallel manner (Fig. 3b). The antiparallel topology of thanatin also negates formation of larger aggregated structures in LPS micelle. The dimeric interface of thanatin sustained by aromatic residues Y10 (Y10′) and hydrophobic residues M21 (M21′) packing interactions across the two β-strands (Fig. 3b). These hydrophobic residues and potentially their mutual packing interactions appear to be important for the antibacterial activity and LPS interactions. As seen, mutated analog peptide Y10M21AA demonstrated diminished antibacterial activity, limited ability of surface charge neutralization and perturbation of LPS structure (Fig. 6). Furthermore, in the dimeric structure of thanatin, the extended N-terminus assumed more defined conformations and folded back along the β-sheet (Fig. 3c, Supplementary Figure 6). These structural changes have yielded two apposite large cationic surfaces in the dimeric structure of thanatin (Fig. 3e). Note, analyses of fluorescence and ITC data using dimer based peptide/LPS interactions were not successful, since the stoichiometric association between LPS micelle and thanatin is not well defined. Regardless, NMR studies further demonstrate that tr-NOESY is a sensitive method for detection of intermolecular NOEs of AMP ligands in complex with LPS micelle. NMR (^31^P, STD), FITC and dansyl fluorescence and MD simulations studies indicated that thanatin assumed a surface localization with LPS micelle or bilayer interacting with the sugar and phosphates at the head group of LPS. MD simulations studies in LPS-DPPE bilayer demonstrated dimeric thanatin can interact with multiple LPS molecules using more than one binding mode or dynamical in nature. Thanatin interactions were predominantly observed with the sugar and phosphate head groups of LPS molecules. The basic residues e.g. K3, K4, R13, R14 and K17, belonging to the cationic patches were found to be primarily involved in LPS recognition (Fig. 5c and d). Antibacterial activity and biophysical studies of the analog peptide R13R14AA indicated critical involvement of residues R13 and R14 (Fig. 6). The atomic-resolution structure of thanatin solved in complex with LPS micelles thus provides mechanistic insights towards bacterial cell agglutination. The antiparallel topology of the dimeric structure of thanatin would potentially allow binding of two or more LPS molecules at the distal ends. The ability of the dimeric thanatin structure to interact with multiple LPS molecules may permit bacterial cells proximity. Further, surface charge neutralization and perturbation of LPS-outer membrane integrity may perhaps facilitate cell-cell associations and agglutination. Taken together, the atomic resolution structures and mode of interactions of thanatin presented in this work can be potentially used for further development of novel cell agglutinating antimicrobial agents.

## Materials and Methods

Thanatin, thanatin Y10AM21A and dansylated thanatin were synthesized commercially by GL Biochem (China). Thanatin R13AR14A was synthesized commercially by KareBay Biochem (China). *E.coli* O111:B4 LPS and *E.coli* 055:B5 FITC-LPS were purchased from Sigma Aldrich. BODIPY TR Cadaverine was purchased from Thermo Fisher Scientific. NMR reagents like DSS, D_2_O were purchased from Cambridge Isotope Laboratories, Inc (Massachusetts, USA).

### Dansyl Cadaverine experiment

To study the binding affinity of the peptides to LPS, peptides were dansylated at the N-terminus and the fluorescence measurements were carried out with Cary Eclipse fluorescence spectrophotometer (Varian inc). 50 μM dansylated peptides were excited at 330 nm wavelength and their emission fluorescence were monitored from 450–650 nm followed by the addition of increasing concentration of LPS. ΔF_max_ was calculated as (F − F_o_)/(F_max_ − F_o_) and was plotted against concentration of LPS, where, F is the fluorescence intensity at maxima for any LPS concentration. F_o_ is the basal fluorescence intensity at maxima. F_max_ is the fluorescence intensity at maxima for maximum LPS concentration. To calculate the dissociation constant, the plot was fitted using Hill’s equation.

### Bodipy Cadaverine Displacement Assay

Relative affinities of thanatin and its analogs to LPS were examined in terms of their ability to displace BODIPY TR cadaverine (BC) from LPS-BC mixture. BC has a high affinity to LPS and its displacement from LPS by the peptides results in dequenching of its fluorescence. 5 μM BC and 50 μg/ml LPS mixture was prepared in 50 mM Tris buffer, pH 7.4. In a 96-well plate, 50 μl of peptides were prepared at final concentrations ranging from 500 μM to 0.01 μM by serially diluting in 50 mM Tris buffer, pH 7.4. Another 50 μl of the aforementioned LPS-BC mixture was added to the wells and fluorescence was measured by exciting samples at 580 nm and emission was recorded from 604–650 nm. BC occupancy factor was calculated as: BC Occupancy Factor = (F_max_ − F)/(F_max _− F_min_) Where, F = Fluorescence intensity at maxima at a particular mediator concentration, F_max_ = Fluorescence intensity at maxima at maximum mediator concentration, F_min_ = Fluorescence intensity at maxima at minimum mediator concentration. Occupancy factor was plotted against concentration of peptides and ED_50_ was calculated using the following equation: $y=1-B+\frac{T-B}{\{1+{(\frac{x}{ED50})}^{-H}\}}$ Where, B is the minimum occupancy factor and T is the maximum occupancy factor.

### Antimicrobial Assay

Minimum Inhibitory Concentration (MIC) of thanatin and analogs was measured using broth dilution method. Mid-log phase cultures of four Gram-negative (*Escherichia coli*, *Pseudomonas aeruginosa* ATCC 27853, *Klebsiella Pneumoniae* ATCC 13883, *Salomonella enterica* ATCC 14028) and four Gram-positive (*Bacillus subtilis*, *Staphylococcus aureus* ATCC 25923, *Streptococcus pyogenes* ATCC 19615, *Enterococcus faecalis* ATCC 29212) bacteria were diluted in MHB to a final concentration of 5 × 10^5^ CFU/ml. In a 96-well plate 50 μl of peptides at final concentrations ranging from 25 μM to 0.25 μM were prepared and another 50 μl of the diluted bacterial solutions were added to each of the wells. The plate was incubated at 37 °C for 18 hrs. OD_600_ was measured and the concentration at which there is complete inhibition of bacterial growth was recorded as the MIC of the peptide.

### FITC-LPS Fluorescence Experiments

The relative ability of thanatin and its analogs to interact with FITC-LPS micelles was studied by measuring the fluorescence of 500 nM FITC-LPS in 10 mM sodium phosphate buffer, pH 7 followed by addition of increasing concentrations of peptide samples. Fluorescence intensity was recorded at an excitation wavelength of 480 nm and emission wavelength of 500–550 nm. % reduction in intensity with respect to free FITC-LPS was calculated as:

1 $$\frac{Fluorescence\,intensity\,of\,FITC-LPS\,in\,presence\,of\,peptide\,at\,maxima}{Flourescence\,intensity\,of\,free\,FITC-LPS\,at\,maxima}\ast 100$$

### Zeta Potential measurements

*E.coli* grown to mid-log phase in LB media were diluted to an OD_600_ of 0.2. Zeta potential of 700 μl of the bacterial cells was first measured. Furthermore, increasing concentrations of peptides were added and zeta potential was measured. The measurements were made in disposable zeta cells with gold electrodes. For each concentration, a total of 3 measurements of 100 runs each was carried out. The experiments were carried out on a zeta sizer Nano ZS (Malvern Instruments, Worcestershire, UK) equipped with a 633 nm He laser.

### Isothermal Titration Calorimetry

The thermodynamic aspect of the peptides binding to LPS was studied using a microcal ITC 200 calorimeter. 50 μM LPS in phosphate buffer, pH 7 was loaded into the sample cell and 1 mM peptide stock was loaded into the syringe. The reference cell was filled with buffer. 25 injections of 1.5 μl peptides were made into the sample cell at 25 °C and the stirring speed was set at 900 rpm. The raw data obtained was fitted using single site binding model in Microcal origin 5.0 software to get the association constant (K_a_) and enthalpy change (ΔH). Dissociation constant (K_d_), Gibbs free energy (ΔG)and entropy change (TΔS) were calculated as: K_d_ = 1/K_a_, ΔG = − RT lnK_a_, TΔS = ΔH − ΔG.

### NMR Spectroscopy

All the NMR spectra were recorded on a Bruker DRX 600 spectrometer, equipped with a cryo-probe and pulse field gradients. Data acquisition and processing were performed with topspin software running on Linux workstation. 0.4 mM peptides in aqueous solution containing 10% D_2_O at pH 5 were used to acquire two dimensional TOCSY (total correlation spectroscopy) and NOESY (nuclear overhauser effect spectroscopy) spectra at 298 K. Mixing times of 80 ms for TOCSY and 300 ms for NOESY were used. DSS (2,2-dimethyl-2-silapentane 5-sulfonate sodium salt) was used as an internal reference for chemical shift. NOESY experiments were performed with 400 increments in t_1_ and 64 transients. WATERGATE procedure was used for water signal suppression. A total of 2 K data points were used in t2 and NMR data was analysed in SPARKY (T.D. Goddard and D.G. Kneller, University of California, San Francisco, CA, USA) program. To record a two-dimensional transferred NOESY spectrum, 30 μM of LPS was added to 0.4 mM peptides in aqueous solution contacting 10% D_2_O at pH 5. The experiment was performed with the same parameters as the NOESY experiment except that the mixing time was 75 ms. In order to study the interaction of peptides with LPS, one dimensional ^31^P NMR spectra of 400 μM LPS in water, pH 6 was recorded on a Bruker DRX 400 spectrometer. Following this, a series of one-dimensional ^31^P NMR spectra were recorded for LPS:peptide ratios of 4:1, 2:1 and 1:1. Two-dimensional STD TOCSY spectra of thanatin-LPS complex in D_2_O, pH 5 were recorded with 330 t_1_ increments and 72 transients using MLEV-17 spin lock field of 80 ms. LPS was saturated at −2.5 ppm (on resonance) and 40 pm (off resonance) for 2 s. Subtraction of the two spectra by phase cycling leads to the required STD spectra. Saturation transfer was achieved by using 40 selective Gaussian 270 pulses with a duration of 50 ms. Relaxation delay of 2.1 s was used. One dimensional STD experiments were carried out by saturating LPS at −3 ppm (on resonance) and 40 ppm (off resonance) with a series of 40 selective Gaussian- shaped pulses (49 ms each) with 1 ms interval leading to a total saturation time of 2 s.

### NMR-Derived Structure Calculations

3-D structures of thanatin and its analogs were calculated using CYANA program. Distance restrains were estimated from two dimensional NOESY spectra on the basis of NOE intensities. Strong, medium and weak NOEs were translated to upper bound distance limits of 2.5, 3.5 and 5.0 Å, respectively. While calculating structure, a disulphide bond constraint was used between cys11 and cys18. SHIFTY was used to calculate the dihedral angle constraint using Hα chemical shift deviation of individual amino acids. Calculations of dimeric structure of thanatin were carried out by adding eleven gly residues connecting two thanatin molecules. Out of 100 structures calculated, 20 lowest energy structures were kept for further analysis. PROCHECK^67^ was used to produce Ramachandran plot, which was in turn used to validate the structures calculated.

### Molecular Dynamics (MD) Simulation

We performed all atom molecular dynamics simulations based on a periodic boundary condition (PBC) box with 120 1,2-Dipalmitoyl-sn-glycero-3-phosphoethanolamine (DPPE) (in lower leaflet), 48 LPS (in upper leaflet) molecules and the thanatin dimer, in a 84 × 84 × 120 cubic box with around 11000 TIP3P water molecules and 192 Ca^2+^ ions, as well as Cl^−^ ions. This LPS/DPPE hybrid bilayer lipid initial model was adopted and modified from the long equilibrium lipid model by Kirschner *et al*.^68^ The schematic diagram of LPS model used in this study has been depicted in Supplementary Fig. 9. The Glycam based force field was used for lipid system. As for two thanatin dimer, NMR model was utilized for simulations, using Amber 99SB-ILDN force field. We adopted the Amber99SB force field parameters for TIP3P explicit water model and ions in all the simulations. Once the simulation boxes were constructed, the systems went through a 1000-steps energy minimization to eliminate possible close contacts among atoms, followed by 100 ps equilibrium in NVT ensemble with restraints added to the heavy atoms of DPPE and LPS molecules. 10 ns equilibrium was employed in NPT ensemble simulations with similar restraints akin to NVT equilibration. Thanatin dimer was randomly inserted into the simulation box replacing water molecules. At 310 K, 100 ps NVT and 10 ns NPT equilibriums, heavy atoms of the thanatin dimer were also imposed by position restraints of 1000 kJ/(mol.nm2). Finally, three repeat runs for 400 ns each, with different initial velocities, were carried out under temperature 310 K. All the simulations were performed with Gromacs package together with Plumed package. The time step for all simulations was 2 fs, while the coordinate data were stored every 2 ps. In NVT and NPT ensembles, temperature coupling was realized using velocity rescaling thermostat scheme updating velocities every 0.1 ps, whereas in NPT ensemble simulations, semi-isotropic pressure coupling method Parrinello-Rahman was utilized to maintain the lipid bilayer structure. SHAKE algorithm and LINCS algorithm were applied, to restrict the bonds between hydrogen-heavy atoms and the heavy-heavy atoms covalent bonds, respectively, to an equilibrium value. Furthermore, PME scheme enabled the long range electrostatic interactions. For both long range electrostatic and van der Waals interactions, the distance cutoff was 1.2 nm. During simulations, dimeric structure of thanatin was maintained by applying eight distance restraints using a quadratic bias potential (force constant 1000) according to Gromacs manual 5.0. It may be noted that structure of dimeric thanatin in water is not favourable due to repulsion among cationic sidechains. The dimer structure is only stabilized in complex with LPS. The minimum distance and center of mass distance between LPS and thanatin dimer were calculated using Gromacs g_mindist and g_dist respectively. The coordination number (*CN*) of LPS/thanatin was defined as

2 $$CN=\sum _{i=1}^{{N}_{LPS}}\sum _{j=1}^{{N}_{than}}C({d}_{ij})$$

and

3 $$C({d}_{ij})=\{\begin{array}{c}1,\,{d}_{ij}\le cutoff\\ 0,\,{d}_{ij} > cutoff\end{array}$$

whereas, N_LPS_ and N_than_ are the total number of atoms in LPS and thanatin dimer respectively, and dij is the distance between atom i in LPS and atom j in thanatin dimer, a distance cutoff = 0.4 nm was used. The thanatin-LPS residue contact probability matrix ($NRe{s}_{LPS}\times NRe{s}_{than}$) was calculated using a distance cutoff = 0.4 nm by counting and normalizing through all the frames in the trajectories, whereas NRes_LPS_ = 15 and NRes_than_ = 42 were the number of residues in the LPS and the thanatin dimer. For each frame of thanatin-LPS complex, 0 or 1 was assigned to each bin according to equation (3) with cutoff = 0.4 nm to have a contact matrix for all residue-residue pairs, and all the matrices summed up and normalized by dividing by the total number of frames.

### Availability of materials and data

All data generated or analysed during this study are included in this published article (and its Supplementary Information files).

## Electronic supplementary material

Dataset 1
