# Supplementary material for: Structure and Interactions of A Host Defense Antimicrobial Peptide Thanatin in Lipopolysaccharide Micelles Reveal Mechanism of Bacterial Cell Agglutination
Source: Sci Rep. 2017 Dec 19;7:17795. doi: 10.1038/s41598-017-18102-6 (PMC5736615; doi:10.1038/s41598-017-18102-6)
Supplement: Supplementary file 1 — Dataset 1 [file 41598_2017_18102_MOESM1_ESM.doc]

**Structure and Interactions of Host Defense Antimicrobial Peptide Thanatin in Lipopolysaccharide Micelles Reveal Mechanism of Bacterial Cell Agglutination**

**Sheetal Sinha^1,2,4^, Liangzhen Zheng^1^, Yuguang Mu^1*^ and Wun Jern Ng^3^, Surajit Bhattacharjya^1^***

**^1^School of Biological Sciences, Nanyang Technological University, 60 Nanyang Drive, Singapore 637551**

**^2^Advanced Environmental Biotechnology Centre, Nanyang Environment and Water Research Institute, Nanyang Technological University, 1 Cleantech Loop, Singapore 637141**

**^3^Division of Environmental and Water Resources, School of Civil and Environmental Engineering, Nanyang Technological University, 50 Nanyang Avenue, Singapore 639798, Singapore and Nanyang Environment and Water Research Institute (NEWRI), Nanyang Technological University, 1 Cleantech Loop, Singapore 637141**

**^4^Interdisciplinary Graduate School, Nanyang Technological University, 50 Nanyang Avenue, Singapore 639798**

^*^Address correspondence to: Surajit Bhattacharjya, 60 Nanyang Drive, Singapore, 637551, e-mail: [surajit@ntu.edu.sg](mailto:surajit@ntu.edu.sg), Fax: 65-6791-3856

^*^Address correspondence to: Yuguang Mu, 60 Nanyang Drive, Singapore, 637551, e-mail: ygmu@ntu.edu.sg, Fax: 65-6791-3856

**Supplementary Figure 1**


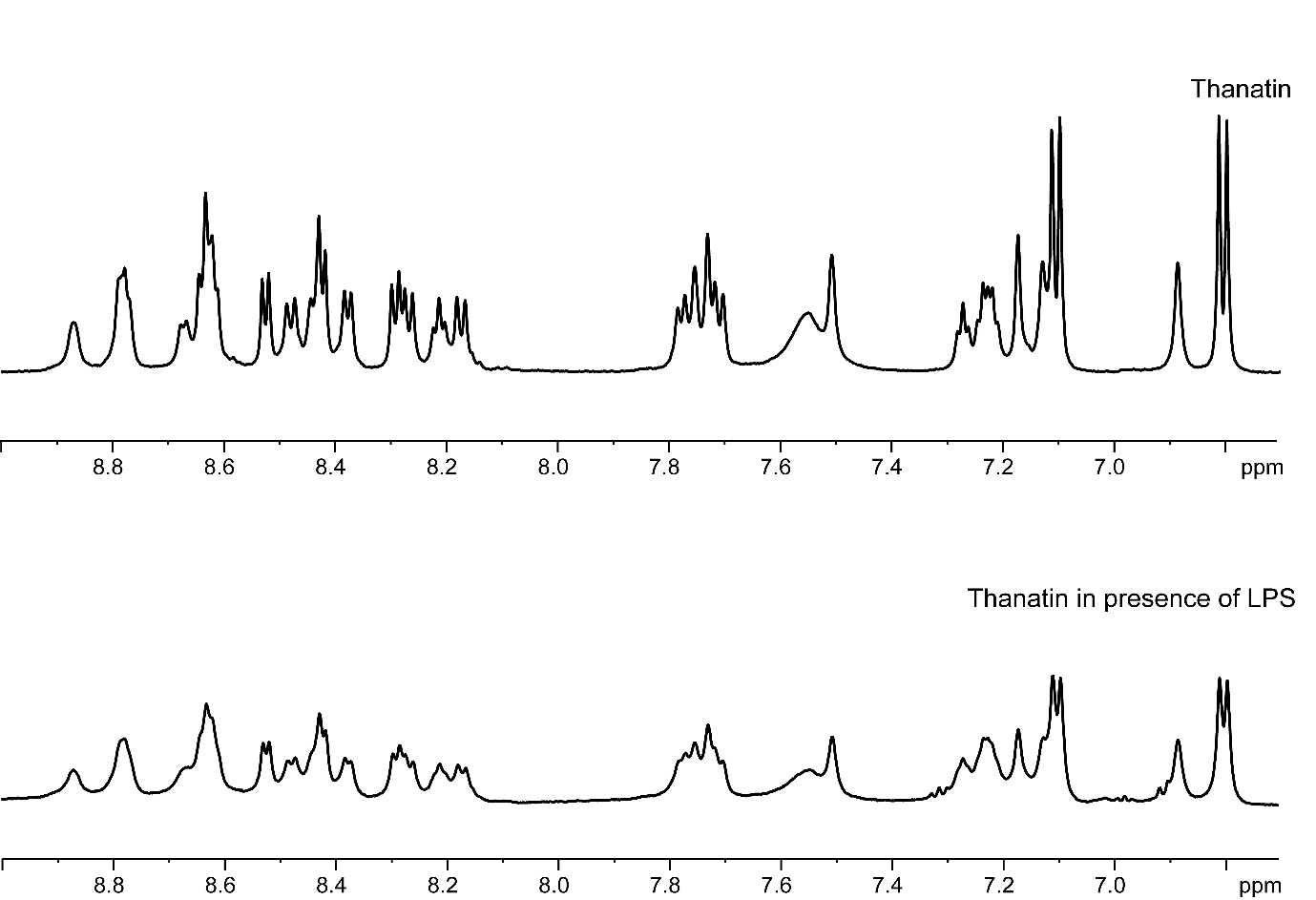


**Supplementary Figure 1|** One dimensional ^1^H NMR spectra, showing down field shifted aromatic and amide proton resonances, of 0.4 mM thanatin in free solution and in presence of 30 μM LPS at 298 K, pH 5. Resonance perturbation in presence of LPS indicates a fast chemical exchange.

**Supplementary Figure 2**


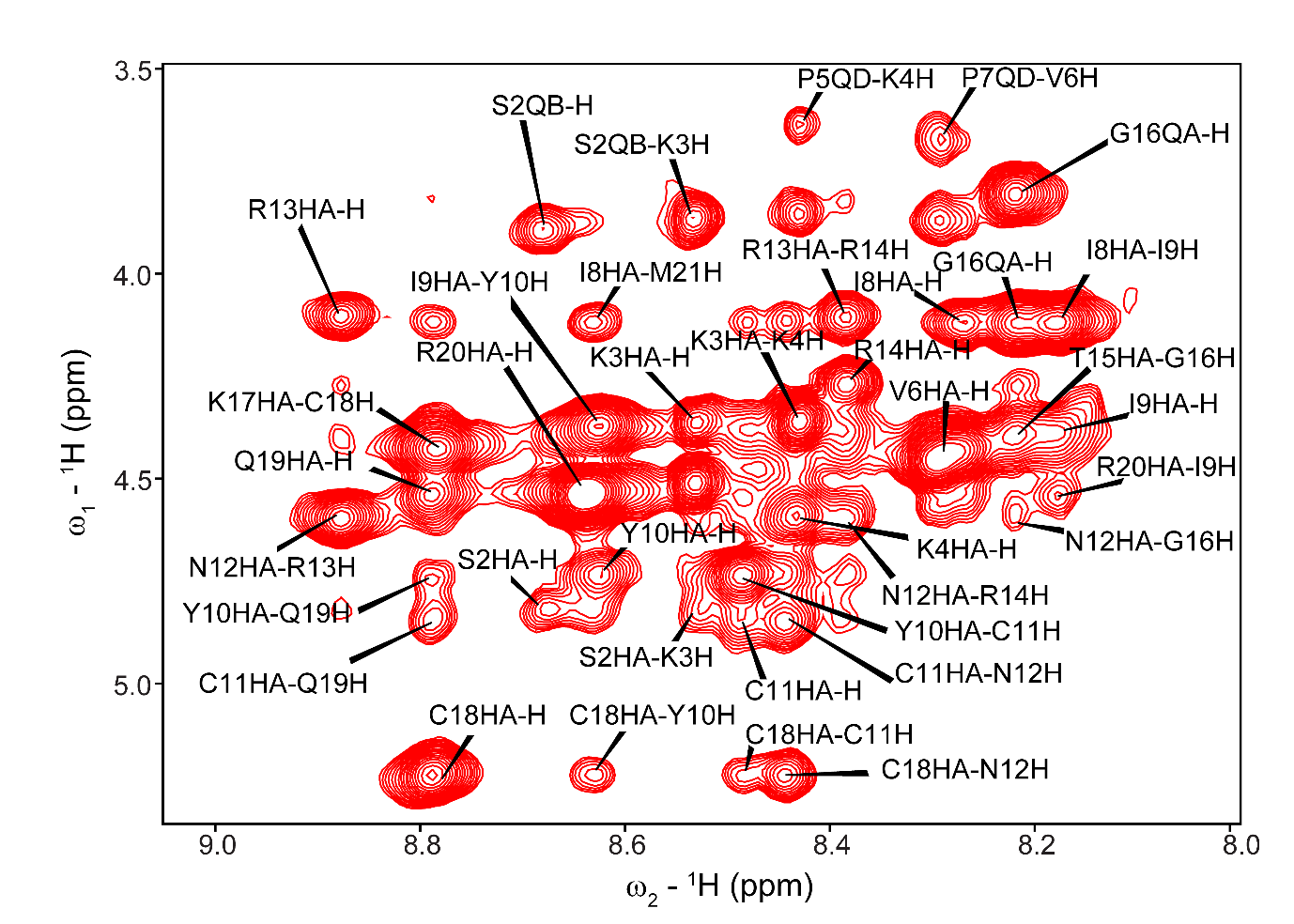


**Supplementary Figure 2|** Finger print region of the two dimensional tr-NOESY spectrum of thanatin showing NOEs involving NH protons with CαH protons.

**Supplementary Figure 3**


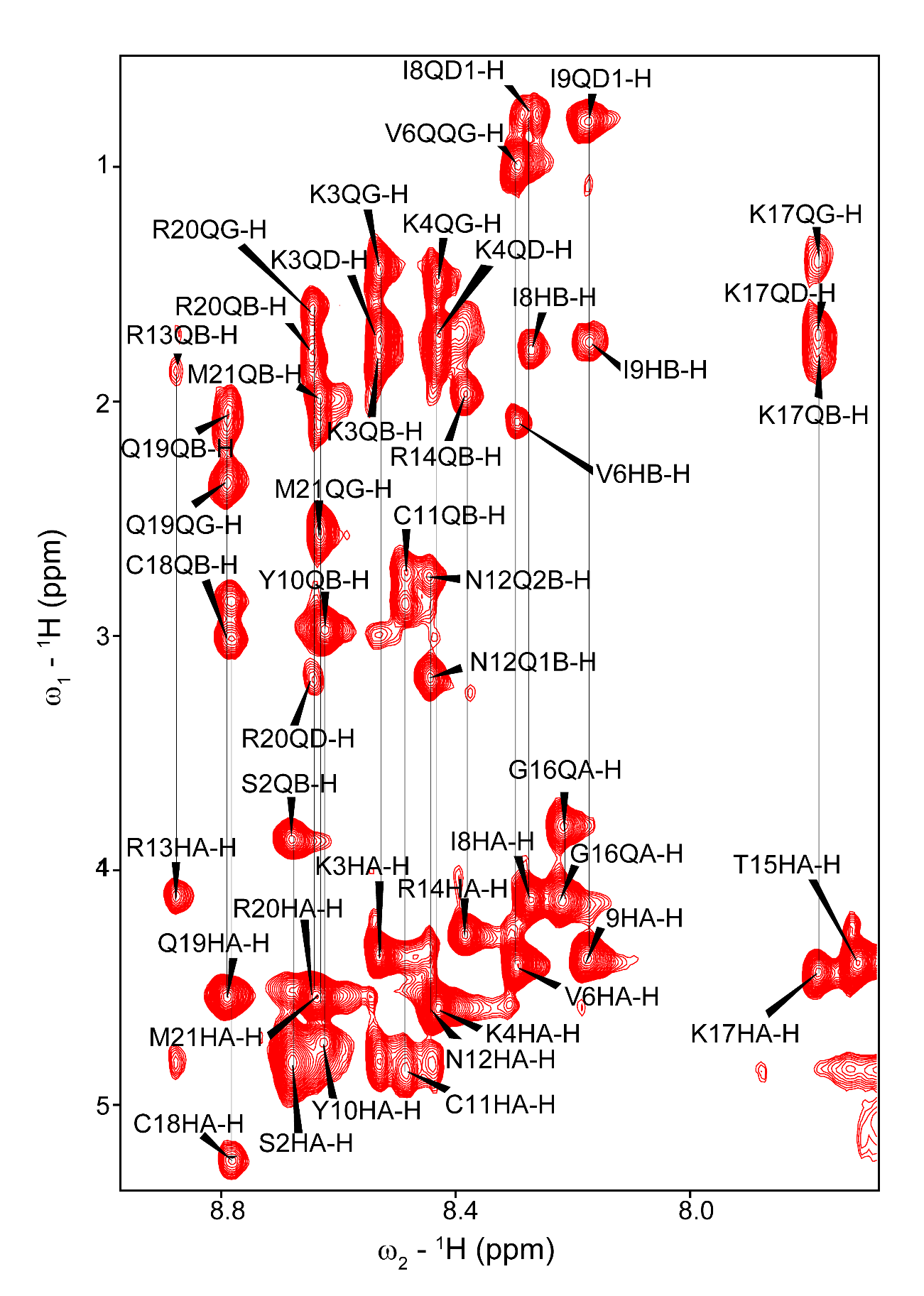


**Supplementary Figure 3|** Section of two-dimensional TOCSY spectrum of thanatin in LPS micelles showing through bond correlations from NH protons to CαH and sidechain protons, for individual amino acid spin system. The TOCSY correlation from NH to C^γ^H_3_ of T15 was not detectable.

**Supplementary Figure 4**


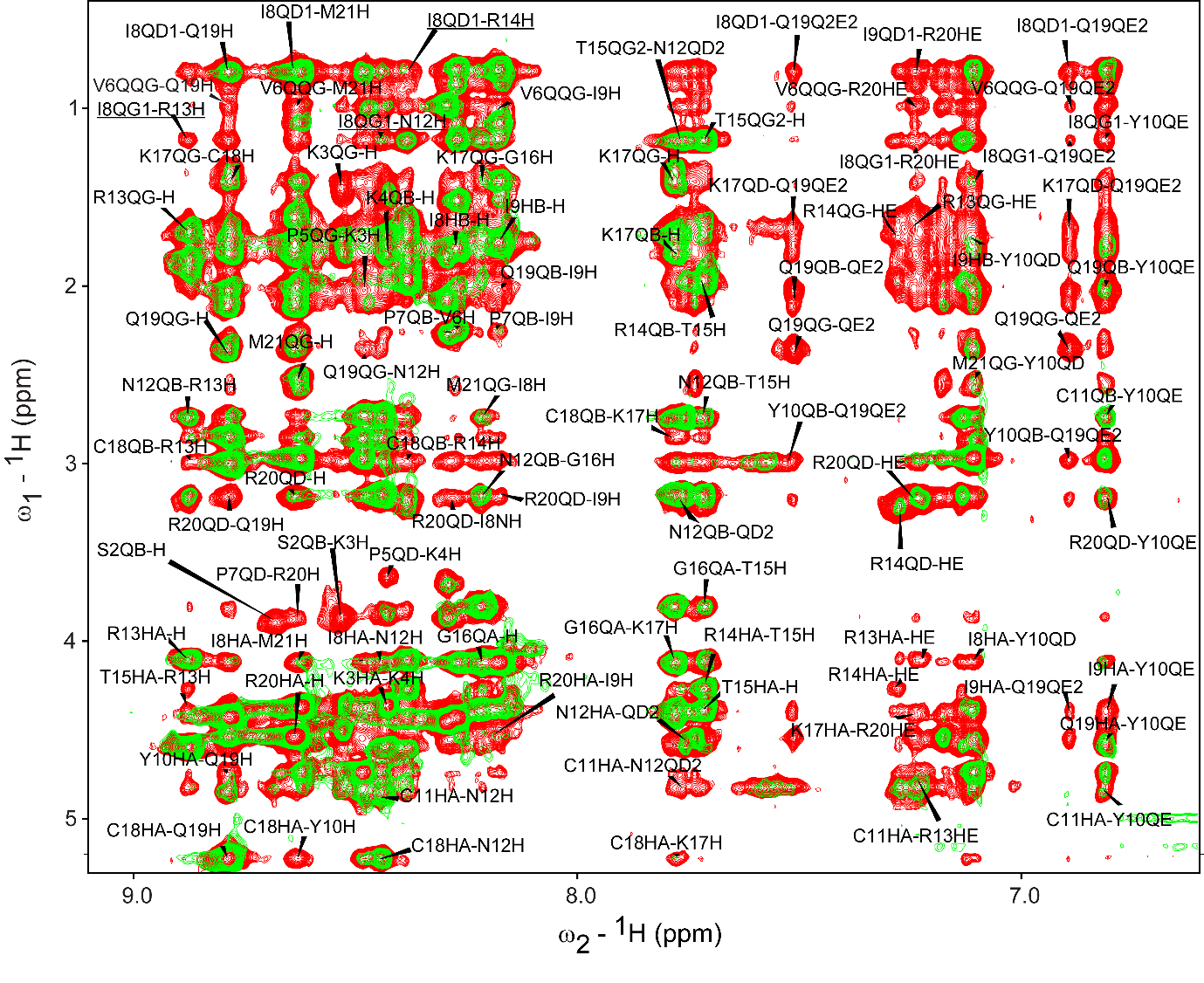


**Supplementary Figure 4|** Two dimensional tr-NOESY spectrum of thanatin in presence of LPS (red) has more long range NOE connectivities as compared to two dimensional NOESY spectrum of free thanatin (green). Furthermore, certain unique NOE cross peaks (underlined) which are present only in tr-NOESY spectrum of thanatin/LPS complex are incompatible to the β-hairpin structure of monomeric thanatin and are used as inter-monomeric NOEs for structure determination.

**Supplementary Figure 5**

**
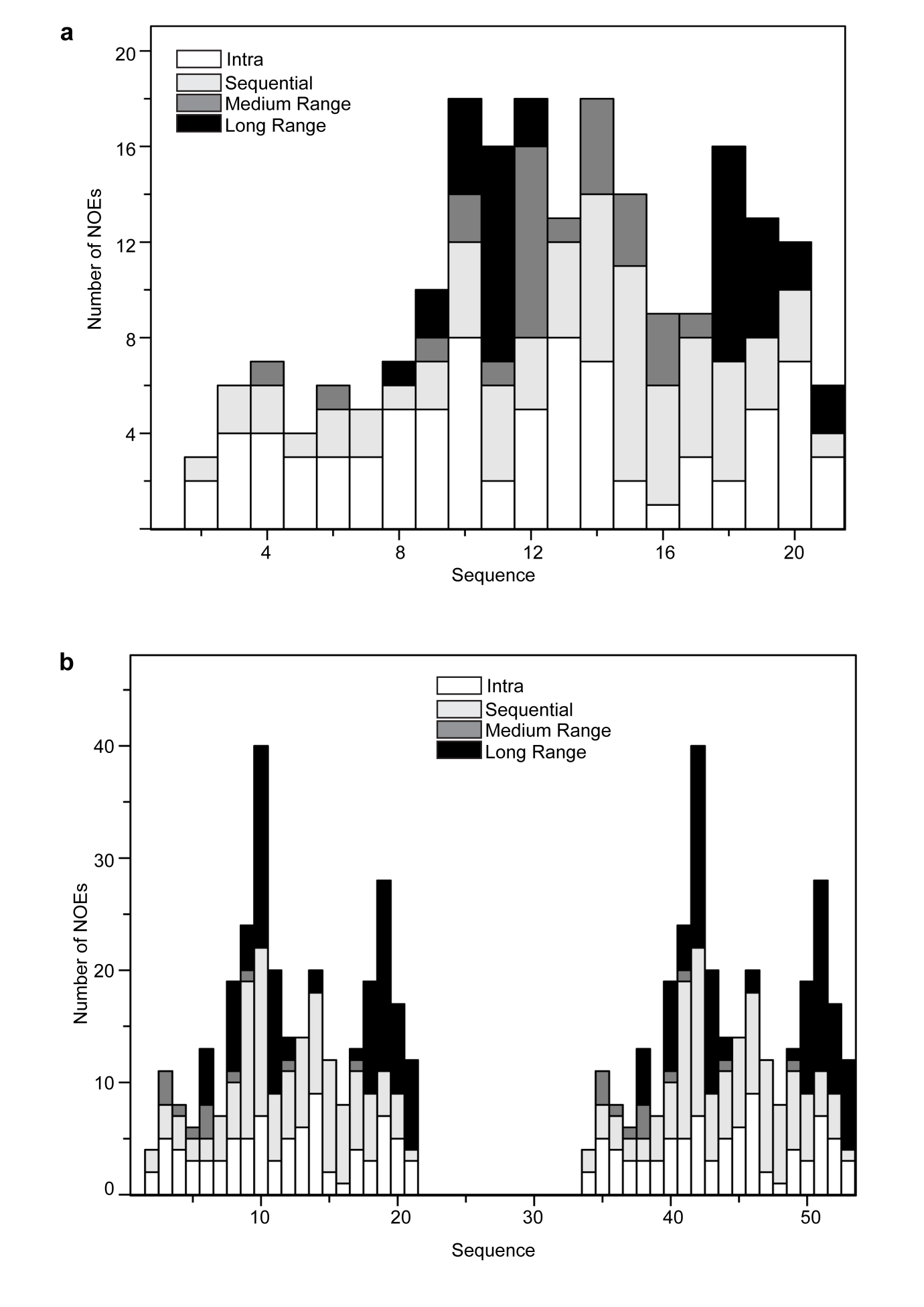
**

**Supplementary Figure 5|** Bar diagrams showing number and type of NOEs for **a,** free thanatin and **b,** thanatin in presence of LPS. Thanatin/LPS complex has more long range NOEs than free thanatin. The gap in the x-axis results from gly-linker residues used to determine the dimeric structure in CYANA (see materials and methods)


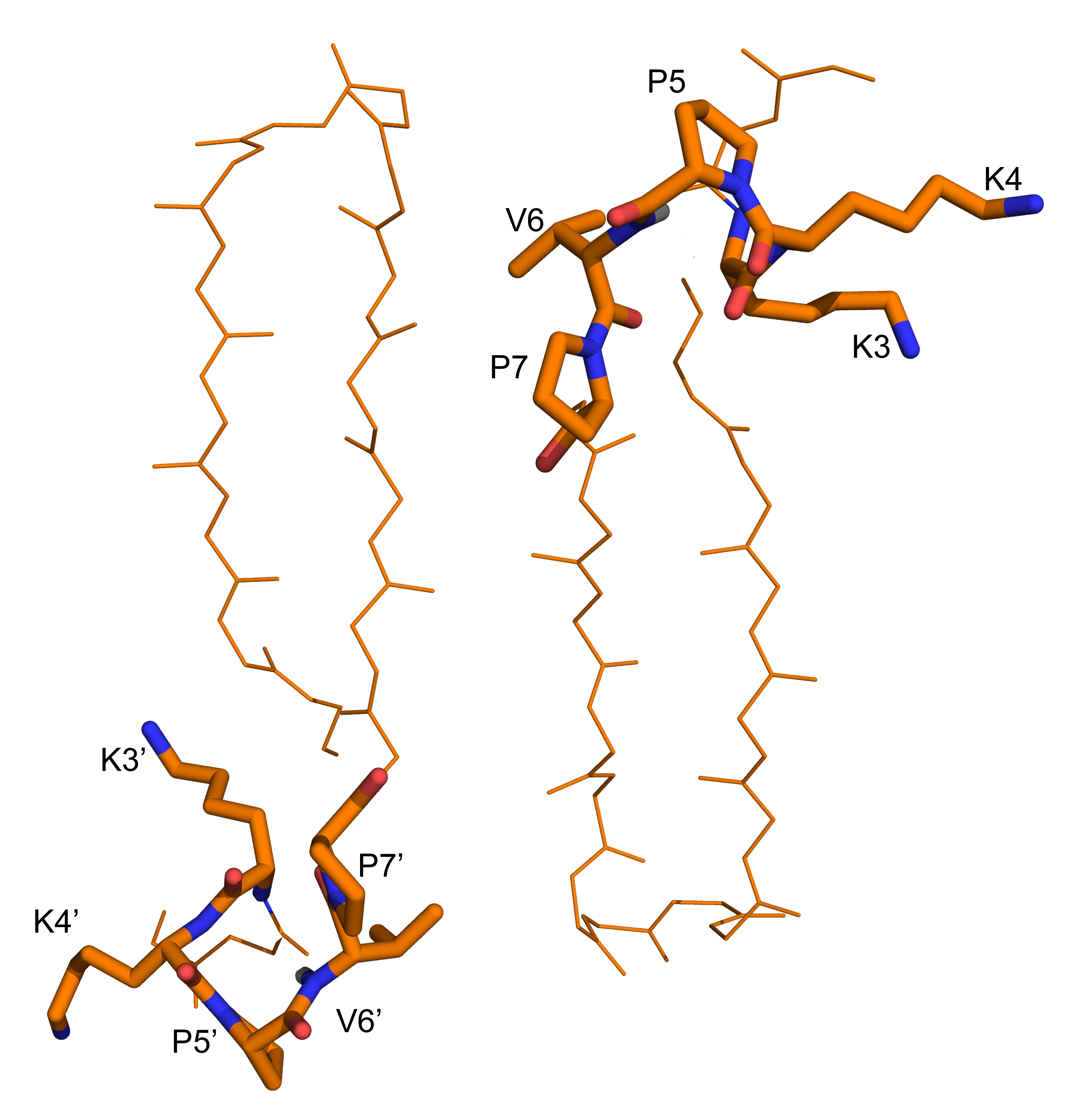
**Supplementary Figure 6**

**Supplementary Figure 6|** N-terminal segment of thanatin folds back toward the β-hairpin structure to adopt a β-turn conformation centring residues K4 and P5 at i+1 and i+2 positions.

**Supplementary Figure 7**

**
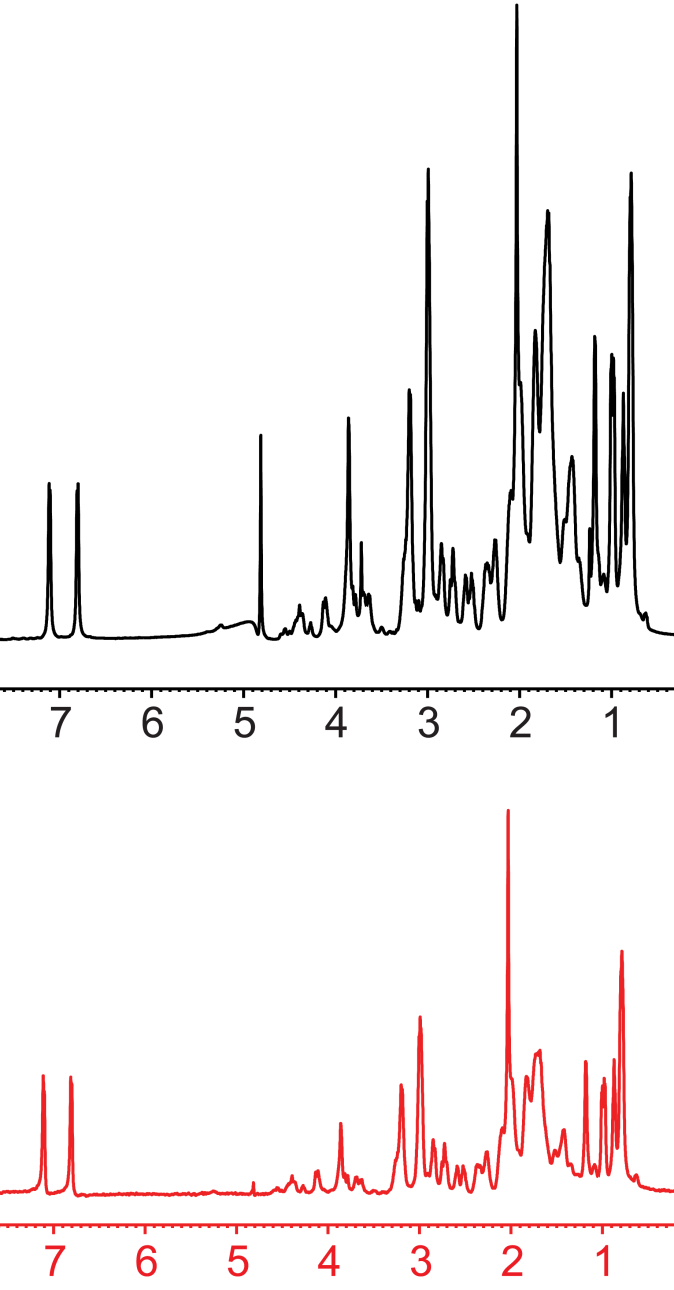
**

**Supplementary Figure 7|** One-dimensional ^1^H STD spectrum (lower panel) and the reference spectrum (top panel) of thanatin in presence of LPS.

**Supplementary Figure 8**

**
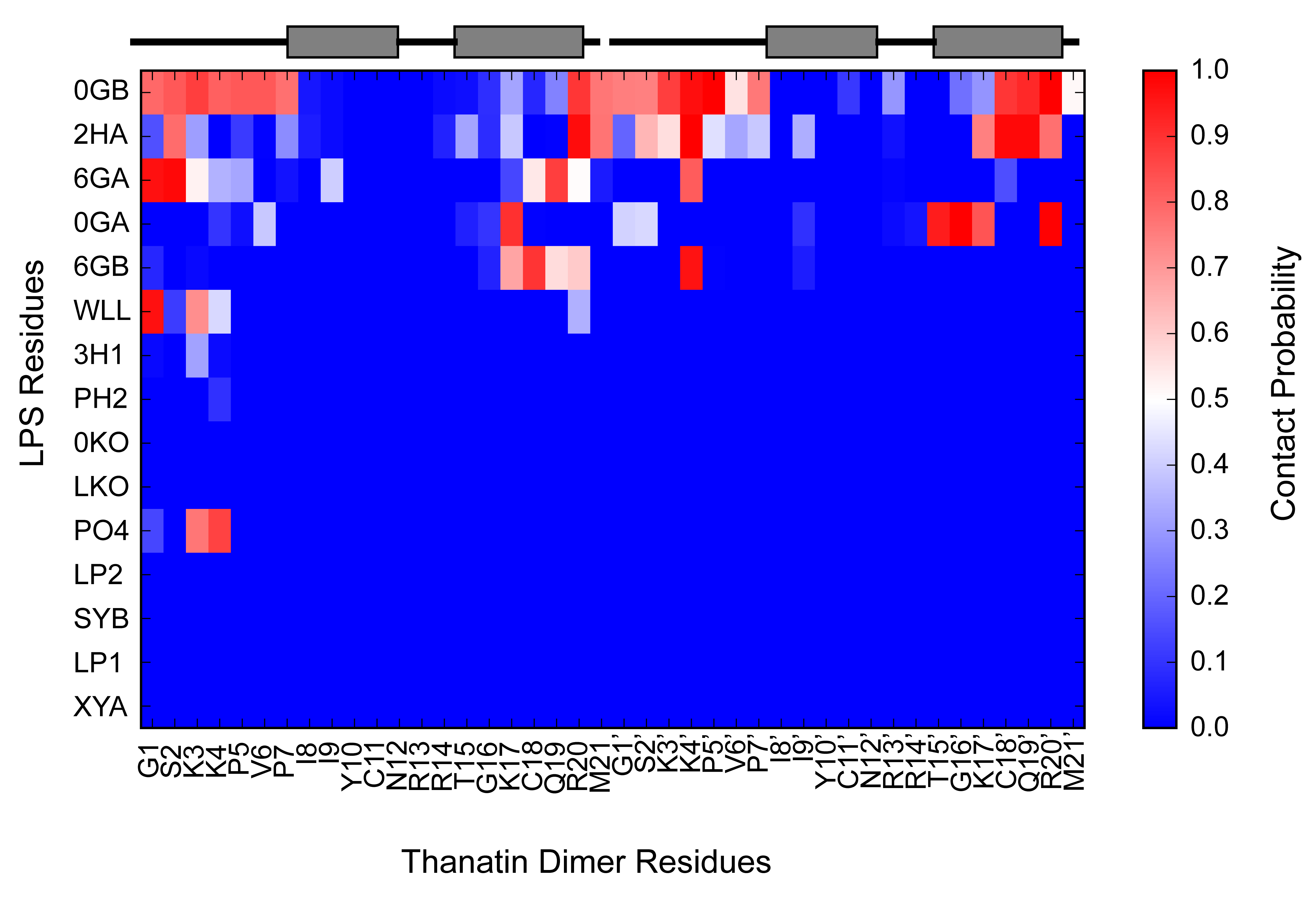
**

**Supplementary Figure 8|** Contact probability of each residue in dimeric thanatin with various LPS residues indicates that N terminal residues of both subunits interact with the LPS along the residues at the middle turn. The secondary structures of thanatin have been shown at the top of the panel, thick bars (in light black) represent β-strands whereas other structural elements are in thin black line.

**Supplementary Figure 9**

**
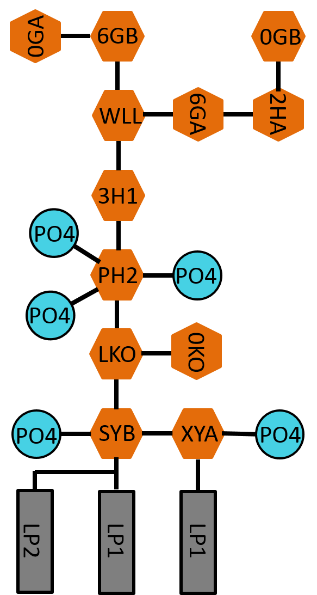
**

**Supplementary Figure 9|** Schematic diagram of LPS used in MD simulation and contact plots. The sugar rings are in orange, phosphate groups are in cyan and the fatty acyl chains are in grey. XYA  2，6-D-acetylglucosamine alpha, SYB  2，3，6-D-N-acetylglucosamine beta, 0GB  terminal D-glucopyranose beta, 0GA terminal D-glucopyranose alpha, 3H1 3-D-rhamnopyranose, PH2 2，3，4，6-D-rhamnopyranose, 0KO terminal 2-Keto-3-deoxy octulonic acid, 6GA 6-D-glucopyranose alpha, 6GB 6-D-glucopyranose beta, 2HA 2-D-rhamnopyranose alpha, LKO 2-keto-3-deoxy octulonic acid, WLL 3，4-D-galactopyranose L form, LP1 two acyl C11 fatty acid chains, LP2 acyl C10 fatty acid chain, PO4 phosphate group

**Supplementary Table S1| Chemical shift of amino acid residues of thanatin in aqueous solution in the presence of LPS micelles**

|  | NH | Hα | Hβ | Hγ | Hδ | Others |
| --- | --- | --- | --- | --- | --- | --- |
| GLY 1 |  | 4.51 |  |  |  |  |
| SER 2 | 8.68 | 4.82 | 3.89 |  |  |  |
| LYS 3 | 8.53 | 4.36 | 1.82 | 1.45 | 1.737 | εCH_2_: 2.99 |
| LYS 4 | 8.43 | 4.59 | 1.82 | 1.48 | 1.712 |  |
| PRO 5 |  | 4.59 | 2.28 | 2.01 | 3.64, 3.85 |  |
| VAL 6 | 8.29 | 4.45 | 2.07 | 0.98 |  |  |
| PRO 7 |  | 4.41 | 2.25, 1.80 | 2.02 | 3.88, 3.69 |  |
| ILE 8 | 8.27 | 4.12 | 1.79 | 1.18, 1.52 | 0.791 |  |
| ILE 9 | 8.18 | 4.38 | 1.74 | 1.08, 1.41 | 0.79 |  |
| TYR 10 | 8.63 | 4.74 | 2.97 |  |  | 2,6 H: 7.11 3,5 H: 6.18 |
| CYS 11 | 8.49 | 4.84 | 2.72, 2.86 |  |  |  |
| ASN 12 | 8.44 | 4.60 | 2.73, 3.18 |  |  | γNH2: 7.135, 7.763 |
| ARG 13 | 8.88 | 4.10 | 1.86 | 1.70 | 3.193 | εNH: 7.234 |
| ARG 14 | 8.39 | 4.27 | 1.96 | 1.64, 1.72 | 3.21 | εNH: 7.28 |
| THR 15 | 7.72 | 4.39 | 4.39 | 1.18 |  |  |
| GLY 16 | 8.22 | 4.12, 3.81 |  |  |  |  |
| LYS 17 | 7.78 | 4.41 | 1.82 | 1.41, 1.35 | 1.713 |  |
| CYS 18 | 8.78 | 5.22 | 2.89, 2.99 |  |  |  |
| GLN 19 | 8.79 | 4.54 | 2.02, 2.11 | 2.35 |  | δNH2: 6.89,7.81 |
| ARG 20 | 8.64 | 4.54 | 1.77 | 1.62 | 3.19 | εNH: 7.25 |
| MET 21 | 8.63 | 4.54 | 2.01, 2.10 | 2.53, 2.57 |  |  |

**Supplementary Table S2|** A list of long range NOEs that were deemed incompatible with the monomeric structure and were further used as inter-monomeric NOEs for structure calculation.

| **Thanatin dimer in complex with LPS** |
| --- |
| 6 VAL QQG – 11 CYS’ H  14 ARG H – 8 ILE’ QD1  6 VAL QQG – 11 CYS’ H  8 ILE QD1 – 13 ARG’ H  8 ILE QD1 – 12 ASN’ H  6 VAL QQG – 14 ARG’ H  6 VAL’ QQG – 11 CYS H  14 ARG’ H – 8 ILE QD1  6 VAL’ QQG – 11 CYS H  8 ILE’ QD1 – 13 ARG H  8 ILE’ QD1 – 12 ASN H  6 VAL’ QQG – 14 ARG H |

**Supplementary Table S3|** Summary of structural statistics of thanatin in complex with LPS micelle

| **Distance constraints** |  |
| --- | --- |
| Sequential [|i-j|= 1] | 118 |
| Medium range [1 <|i-j|< 4] | 8 |
| Long range [|i-j|≥ 4] | 78 |
| Inter monomer  Total | 10  386 |
|  |  |
| **Dihedral-angle constraints** | 64 |
|  |  |
| **Deviation from mean structure (Å)** |  |
| All backbone atoms | 1.42 |
| All heavy atoms | 1.93 |
|  |  |
| **Ramachandran plot for the mean structure (%residues)** |  |
| in most favoured region | 75 |
| in additionally allowed region | 25 |
| in generously allowed region | 0 |
| in disallowed region | 0 |
